# Prevalence, risk factors, and outcomes of dysphagia after stroke: a systematic review and meta-analysis

**DOI:** 10.3389/fneur.2024.1403610

**Published:** 2024-07-17

**Authors:** Wenjing Song, Minmin Wu, Haoran Wang, Ruifeng Pang, Luwen Zhu

**Affiliations:** ^1^Heilongjiang University of Chinese Medicine, Harbin, China; ^2^The Second Affiliated Hospital of Heilongjiang University of Chinese Medicine, Harbin, China

**Keywords:** dysphagia, stroke, post-stroke dysphagia, systematic review, meta-analysis, prevalence

## Abstract

**Background:**

Dysphagia is a common complication after stroke, which not only brings adverse outcomes but also greatly affects the quality of life of patients. At present, there is no systematic review or meta-analysis to comprehensively evaluate the epidemiological characteristics of post-stroke dysphagia (PSD). A systematic review of the prevalence, risk factors, and prognosis of PSD is essential.

**Methods:**

Through 31 December 2022, a comprehensive literature search was performed for observational studies related to PSD. Five databases were retrieved. Random-effects models were used to estimate the pooled prevalence, odds ratio (OR), and 95% CIs.

**Results:**

A total of 34 studies were included, and the results showed that the overall prevalence of PSD was 46.6% (95% CI, 0.405–0.528). The prevalence of dysphagia in ischemic stroke and hemorrhagic stroke was 43.6% (95% CI 0.370–0.501) and 58.8% (95% CI 0.519–0.654), respectively. The prevalence of PSD in Africa was 49.4% (95% CI, 0.196–0.792), in Asia was 40.1% (95% CI, 0.348–0.454), in Europe was 45.8% (95% CI, 0.327–0.590), in North America was 44.3% (95% CI, 0.370–0.517), in South America was 57.5% (95% CI, 0.441–0.708), and in Oceania was 64.1% (95%CI, 0.558, 0.724). In risk factor analysis, hypertension, previous stroke, and atrial fibrillation were significantly associated with the occurrence of PSD, pooled OR = 1.179 [(95% CI, 1.002–1.386), *p* < 0.05], pooled OR = 1.514 [(95% CI, 1.204–1.905), *p* < 0.001], and pooled OR = 1.980 [(95% CI, 1.580–2.481), *p* < 0.001]. In outcome studies, the prevalence of aphasia and dysarthria in PSD was 35.6% (95% CI, 0.213–0.499) and 54.5% (95% CI, 0.293–0.798), respectively. The prevalence of respiratory tract infection was 27.1% (95%CI, −0.038–0.579), and the prevalence of pneumonitis was 32.1% (95% CI, 0.224–0.418). Persistence of dysphagia at discharge and at 1 month was 74.5% (95% CI, 0.621–0.869) and 50.9% (95% CI, 0.142–0.876), respectively. Mortality rates for PSD patients during admission and discharge at 1 month, 3 months, and 1 year were 11.8% (95% CI, 0.083–0.152), 26.5% (95% CI, 0.170–0.359), 25.7% (95% CI, 0.19–0.324), and 31.3% (95% CI, 0.256–0.369), respectively.

**Conclusion:**

This study found that the overall prevalence of PSD was 46.6%. Prevalence is most influenced by the diagnosis method. Hypertension, history of stroke, atrial fibrillation, patient age, and stroke severity were risk factors significantly associated with PSD. The prevalence of aphasia, dysarthria, respiratory tract infection, and pneumonitis in PSD patients is 2–4 times that of patients without PSD.

**Systematic review registration**: www.crd.york.ac.uk/PROSPERO, PROSPERO, CRD42021252967.

## 1 Introduction

Stroke is the leading cause of death and disability worldwide, and the 2010 global estimates ranked stroke as the second most common cause of death worldwide (1). Studies have shown that while the incidence of stroke has decreased in high-income countries, it is still increasing in low-income and middle-income countries (2). Dysphagia is a common post-stroke complication and one of the first obstacles to recovery after a stroke. Dysphagia not only increases mortality after stroke but also greatly affects the patient’s quality of life, and it leads to asymmetry of the swallowing musculature in both motor cortices. Stroke affects major swallowing projections of the cerebral hemispheres, leading to dysphagia. This asymmetrical bilaterality may explain why several stroke patients have dysphagia. Recovery from post-stroke dysphagia (PSD) is associated with compensatory changes in the unaffected cerebral hemispheres, which explains the ability of PSD patients to regain safe swallowing function in a relatively short time (3, 4). However, if dysphagia is not detected early and the patient continues to eat, the results can be life-threatening, and serious complications such as pneumonia, dehydration, malnutrition, and asphyxia can occur (5). Moreover, the ability of stroke patients to eat and drink through the mouth has become a key factor in discharge (6).

The prevalence of PSD ranges from 18 to 81% (7–10), which is varied. The reasons for such inconsistencies in prevalence may include different locations of stroke lesions and study areas, and further discussions on these inconsistencies or potential contributing factors are needed. Although several studies have analyzed the epidemiology of PSD, most did not report or explain the factors that may affect the prevalence of PSD, which may further explain the inconsistencies in prevalence. Moreover, there is no consensus on factors that may influence the development of PSD (11, 12), and there has been no comprehensive systematic review of PSD outcomes that included large sample studies. Therefore, we aimed to systematically assess the actual prevalence of PSD and investigate the impact of other factors on PSD and patient outcomes to provide a better and more comprehensive understanding of the epidemiological features of PSD, which can provide a basis for clinical practice.

## 2 Method

### 2.1 Data sources and search strategy

This systematic review and meta-analysis was performed in accordance with the Preferred Reporting Items for Systematic Reviews and Meta-Analyses statement (13) (PRISMA; Supplementary Table 1) and the Meta-Analyses of Observational Studies in Epidemiology guidelines (14) (MOOSE; Supplementary Table 2). The protocol was registered *post-hoc* in the International Prospective Register of Systematic Reviews (PROSPERO CRD42021252967 at www.crd.york.ac.uk/PROSPERO).

A literature search was performed using five electronic medical databases, including Embase, PubMed, Cochrane Library, Web of Science, and MEDLINE (via Web of Science), to identify all articles published on the prevalence of PSD and its related factors or outcomes from their inception to 31 December 2022. Furthermore, to ensure that no relevant studies were missed, we traced references to the full text that had been identified. We used a search strategy that combines subject terms with free words. The detailed literature search strategy for every database is shown in Supplementary Appendix 1.

### 2.2 Inclusion and exclusion criteria

Only articles that used an observational design, including a cross-sectional, case–control, or cohort design, and reported the prevalence or incidence of PSD or had sufficient data to allow the calculation of the prevalence of PSD in the general population or in clinical patients were included in this study. We included only articles published in the English language. Regarding the inclusion of stroke patients who had a clear hospital-based diagnosis of ischemic or hemorrhagic stroke, we excluded studies on patients with transient ischemic attacks (TIAs) and on screening for dysphagia without a clear diagnosis method and the time of diagnosis. Considering that PSD may be related to the recurrence of stroke, type, and location of stroke, we only included studies that reported on ischemic or hemorrhagic stroke and included patients with both hemorrhagic and ischemic strokes and studies in which the prevalence of swallowing disorders could be calculated separately from the given data for patients with a first-time or recurrent stroke. We excluded studies that included only ischemic or hemorrhagic stroke at specific sites, such as stroke in the brainstem only, and studies on first-time stroke only or recurrent stroke only. We excluded review articles, case reports, protocols, brief communications, personal opinions, letters, posters, conference abstracts, or laboratory studies as well as literature for which the prevalence of PSD could not be calculated from the given data or were not available after attempts to contact the authors. If there were duplicate articles on the same cohort within the same period, we included only the articles with the most data. There was no restriction on the year of publication.

### 2.3 Study selection and data extraction

After excluding all duplicates, two researchers (WS and HW) independently screened the titles and abstracts of the articles and subsequently screened the full texts for relevance to the topic. If any article met the inclusion criteria, they were included in the final meta-analysis. Two investigators (WS and MW) independently retrieved information from the included studies and cross-checked the information to ensure the integrity of the content. If inconsistencies or disagreements arose during the screening process or information retrieval, they were resolved by consensus between the two investigators or by consultation with a third and senior investigator (LZ). Data extracted included the name of the first author, year of publication, country, continent, time of study, participants’ age, population origin, type of stroke, stroke lesions, stroke severity, number of dysphagia patients, total study population, diagnosis method and time of PSD diagnosis, severity of dysphagia, risk factors (diseases mentioned in the literature that may be associated with PSD), and outcomes (complications or prognosis of PSD). When multiple time points of dysphagia were diagnosed in the same study, only the time of the first diagnosis was used for the calculation of the main prevalence, and the other diagnosis times were analyzed as the outcomes of dysphagia. We inserted the data into two tables (Table 1 and Supplementary Table 3).

**Table 1:** General characteristics of the included literature.

Author and year	Continents	Study design	Type of stroke	No. of PSD	No. of total	Diagnose method
Marlis 2008 (15)	North America	Case–control	Ischemic	14	29	Clinical
Zahra 2021 (16)	Asia	Cross-section	Ischemic and hemorrhagic	36	100	Clinical
Antia 2019 (17)	Europe	Cohort	Ischemic and hemorrhagic	60	106	Clinical
Eman 2021 (18)	Africa	Cross-section	Ischemic and hemorrhagic	98	250	Clinical
Hamidon 2006 (19)	Asia	Cohort	Ischemic	55	134	Clinical
Michael 2013 (20)	North America	Cohort	Ischemic	25	67	Clinical
Giselle 2000 (21)	Oceania	Cohort	Ischemic and hemorrhagic	82	128	Instrument
Maurizio 2004 (22)	Europe	Cohort	Ischemic and hemorrhagic	141	406	Clinical
Mahsa 2021 (23)	Asia	Cross-section	Ischemic and hemorrhagic	136	349	Clinical
Anna 2018 (24)	Europe	Cohort	Ischemic	81	140	Clinical
Heather 2017 (25)	North America	Cohort	Ischemic	76	160	instrument
Sani 2017 (26)	Africa	Cohort	Ischemic and hemorrhagic	32	94	Clinical
SK 2017 (27)	Europe	Cross-section	Ischemic and hemorrhagic	165	200	Instrument
Bendix 2018 (28)	Europe	Cohort	Ischemic and hemorrhagic	571	687	Instrument
Zeki 2010 (29)	Asia	Cohort	Ischemic and hemorrhagic	41	72	Clinical
Kaila 2019 (30)	North America	Cohort	Ischemic	32	100	Clinical
Shiva 2019 (31)	Asia	Cohort	Ischemic	29	88	Clinical
Elien 2020 (32)	Europe	Cohort	Ischemic	35	151	Clinical
Nayeon 2021 (33)	Asia	Cohort	Ischemic	1940	5,740	Clinical
Danielles 2016 (34)	South America	Cross-section	Ischemic and hemorrhagic	32	42	Clinical
Juli 2019 (35)	South America	Cohort	Ischemic and hemorrhagic	86	201	Clinical
Heather 2013 (36)	North America	Cohort	Ischemic	98	221	Clinical
Shiva 2016 (37)	Asia	Cross-section	Ischemic and hemorrhagic	545	113	Clinical
Smithards 2007 (38)	Europe	Cohort	Ischemic and hemorrhagic	567	1,188	Clinical
Avinash 2019 (39)	Europe	Cohort	Ischemic	81	340	Clinical
Polo 2009 (40)	Europe	Cohort	Ischemic and hemorrhagic	62	151	Clinical
Rofes 2018 (41)	Europe	Cohort	Ischemic and hemorrhagic	178	395	Clinical
Mohamed 2016 (42)	Europe	Cohort	Ischemic	3,083	12,276	Clinical
Carlo 2019 (43)	Europe	Cohort	Ischemic and hemorrhagic	94	249	Clinical
Sachiyo 2021 (44)	North America	Cohort	Ischemic and hemorrhagic	233	427	Clinical
Hakan 1998 (45)	Europe	Cohort	Ischemic and hemorrhagic	14	72	Clinical
Anna 2012 (46)	South America	Cohort	Ischemic and hemorrhagic	134	212	Clinical
Aline 2016 (47)	South America	Cohort	Ischemic and hemorrhagic	50	100	Clinical
Felix 2021 (48)	Europe	Cohort	Hemorrhagic	84	132	Clinical

### 2.4 Quality assessment

Since three types of observational studies were included, we assessed them separately. We used the Newcastle–Ottawa scale (NOS) (49) to assess the risk of bias in the included case–control or cohort studies. The scale was divided into three main parts, including the quality of the selected cohort, the comparability of the cohort, and the adequacy of the outcome or follow-up. The maximum score for each study was 9. Study quality was divided into the following three categories based on quality scores: high (0–4), moderate (5–6), and low risk of bias (7–9). We used the Agency for Healthcare Research and Quality (AHRQ) (50) Scale to evaluate the quality of cross-sectional studies. The AHRQ checklist for evaluation consists of 11 items. Each item is marked as “1” when the answer is “yes” and “0” when the answer is “unclear” or “no.” Studies were rated as high, moderate, and low risk of bias when the quality scores were 0–3, 4–7, and 8–11, respectively. Quality assessments were conducted independently by two researchers (WS and RP), and when inconsistencies or disagreements were encountered, they were resolved in consultation with a third researcher (LZ). The details of the evaluation scale are presented in Supplementary Appendix 2.

### 2.5 Data analysis

We used the logit method to transform the reported prevalence in each study due to the heterogeneity of the studies and subsequently performed an inverse-variance-weighted random-effects meta-analysis using the method by DerSimonian and Laird (51). Next, we obtained the pooled prevalence (95% CI) of PSD in the overall population. Between-study heterogeneity was assessed using the *I*^2^ statistic and the *p*-value for heterogeneity (Cochran’s Q statistic). The range of the *I*^2^ statistic is between 0 and 100%; *I*^2^ ≥ 50% indicates significant heterogeneity (52). The results are displayed using forest plots. To explore the sources of heterogeneity and to obtain the effects of different regions, different stroke types, sex, method and time of diagnosis, and first or recurrent stroke on the prevalence of dysphagia, we first explored the prevalence of PSD separately using subgroup analysis of these variables. If heterogeneity was still not found, we performed a meta-regression analysis using study characteristics, including year of publication, continents, type of study, and method of PSD diagnosis, as moderating variables. To explore exposure factors other than the above subgroups that may influence the occurrence of swallowing disorders after stroke, we extracted data on the effect of other possible exposure factors such as hypertension and diabetes on the prevalence of swallowing disorders recorded and used these data to calculate the crude OR of 2 × 2 tables for each study and provided its 95% CI and a combination of random-effects heterogeneity models to generate pooled estimates to determine the exposure factors for PSD. If the OR could not be calculated, we performed a systematic descriptive analysis. Regarding the outcomes of swallowing disorders, we performed a pooled analysis of the outcomes reported in the literature. We used a random-effects model to pool patients’ mortality, incidence of pneumonia, and other reported comorbidities. Funnel plots and Egger’s test were used to assess publication bias for the outcomes. To ensure that the study results are credible and to eliminate the effects of small population, heterogeneity, or study quality on outcomes, sensitivity analysis was performed to assess the stability of the results in the included literature. The Stata 16 software (Stata Statistical Software: College Station, TX: Stata Corp LP) was used to Meta-analyze the extracted data.

## 3 Results

### 3.1 Literature search and features of included literature

**Figure 1:**
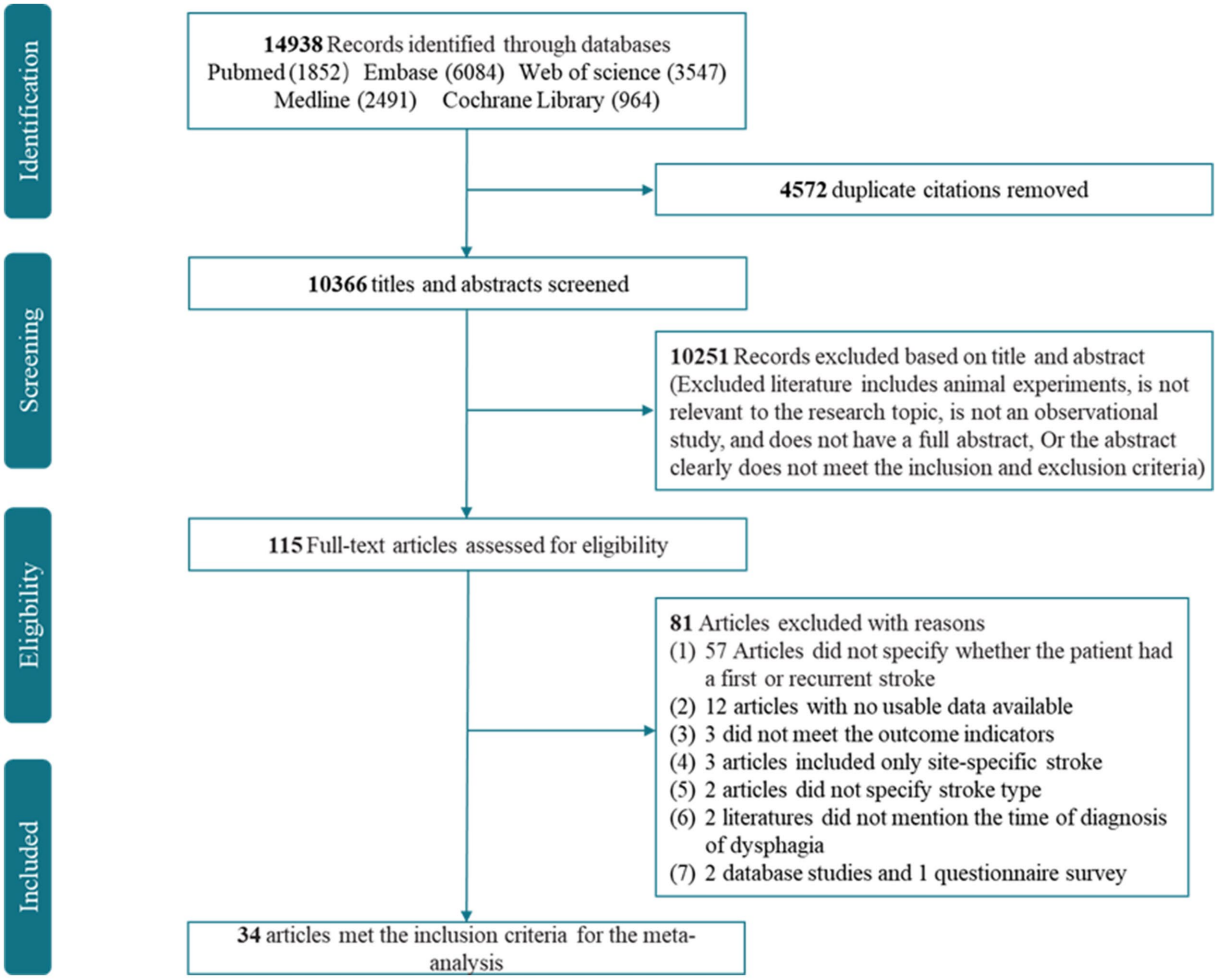
Flowchart of the identification of eligible studies.

We retrieved 14,938 studies from the five databases. After removing 4,572 duplicates, the titles and abstracts of the remaining 10,366 studies were checked, and 10,251 studies were excluded. The full texts of the remaining 115 studies were screened, and 34 studies that met the inclusion criteria were identified (15–48). The detailed screening process is shown in Figure 1.

The detailed study characteristics are shown in Table 1 and Supplementary Table 3. Overall, all included studies were hospital-based studies with publication years ranging from 1998 to 2021, study sample sizes ranging from 29 to 12,276, and a total study population of 25,022 across 17 countries, including Canada, the USA, the UK, Belgium, Spain, Nigeria, Brazil, Sweden, Germany, Italy, Iraq, Egypt, Switzerland, Iran, Korea, Malaysia, and Australia, and 6 continents, of which 7 studies (16, 19, 23, 29, 31, 33, 37) were conducted in Asia, 14 (17, 22, 24, 27, 28, 32, 38–43, 45, 48) in Europe, 6 (15, 20, 25, 30, 36, 44) in North America, 4 (34, 35, 46, 47) in South America, 2 (18, 26) in Africa, and 1 in Oceania (25). All studies reported the prevalence of PSD; 22 studies (15, 18–20, 22, 24–26, 28, 36, 38–48) documented risk factors of PSD, and 23 studies (21, 23, 24, 26, 30–37, 39–52) documented the complications of PSD or mortality of PSD patients. One was a case–control study, 26 were cohort studies, and 7 were cross-sectional studies. The quality assessment of each article included in this study is shown in Supplementary Tables 4, 5. The average NOS score was 5.4, and the average AHRQ score was 6.7, indicating that most studies were of moderate quality.

### 3.2 Prevalence of PSD

**Figure 2:**
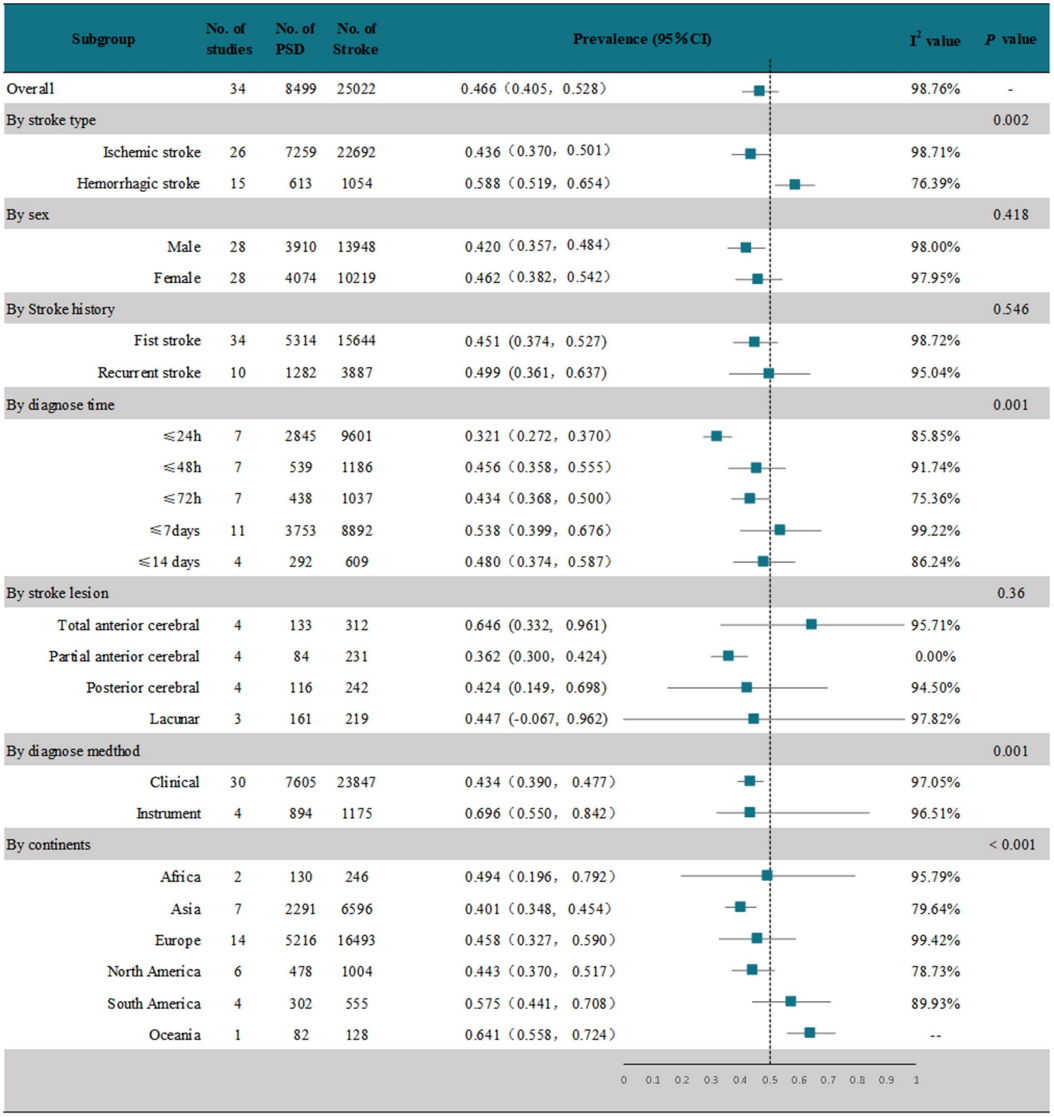
Overall prevalence and subgroup analysis of PSD.

**Figure 3:**
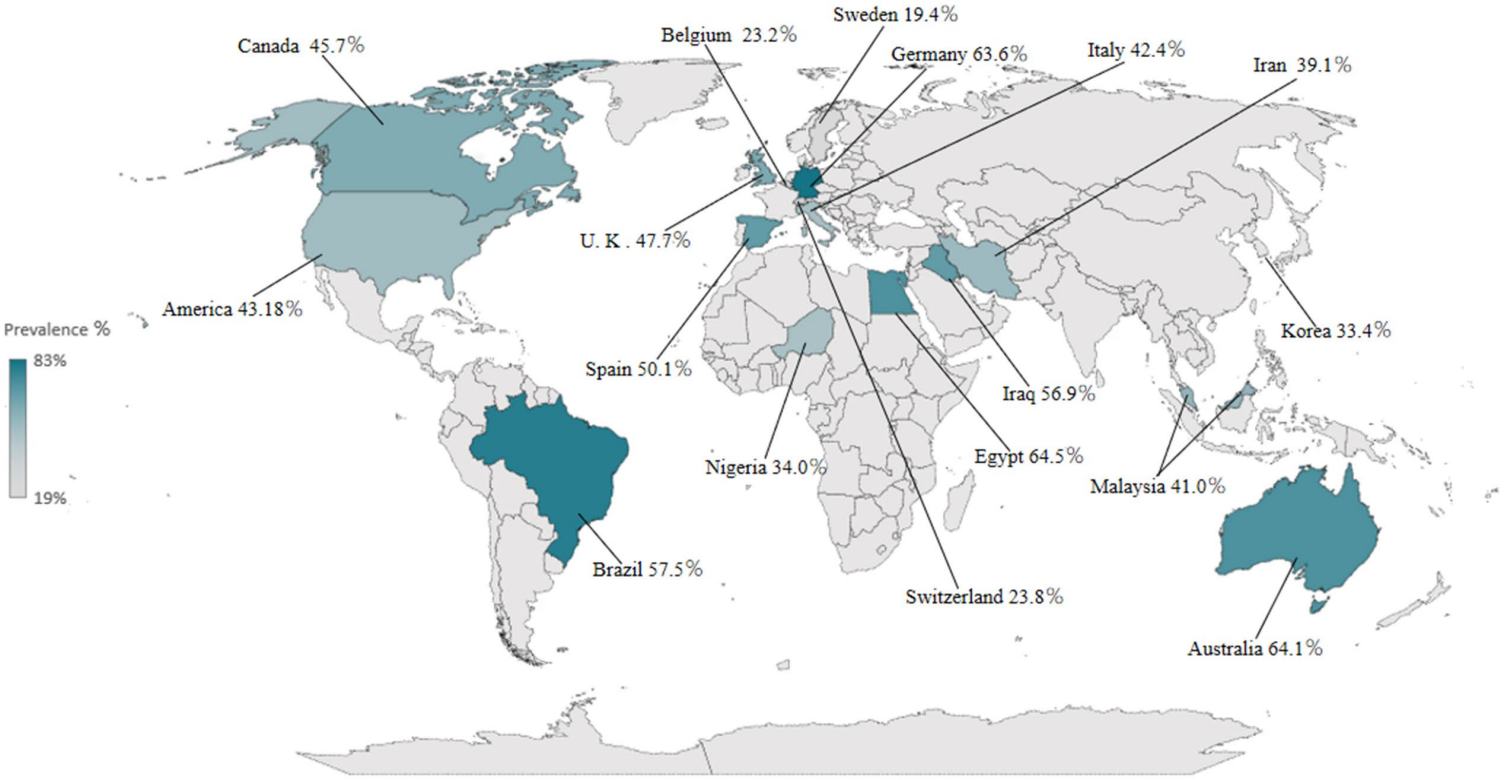
Global prevalence of PSD.

The meta-analysis of 34 studies showed that the random-effects pooled overall prevalence of PSD was 46.6% (95% CI, 0.405–0.528), with high heterogeneity (*I*^2^ = 58.76%; *p* < 0.001) (Figure 2 and Supplementary Figure 1). Figure 3 shows the prevalence of dysphagia among individuals with stroke for all countries with at least one study.

These studies were analyzed in subgroups according to the different stroke types (Figure 2 and Supplementary Figure 2). In 26 studies (15–20, 22, 24, 25, 28–44, 46) that reported dysphagia after ischemic stroke, the pooled prevalence was 43.6% (95% CI, 0.370–0.501), with high heterogeneity (*I*^2^ = 98.71%; *p* < 0.001). In 15 studies (17, 18, 22, 28, 29, 31, 35, 37, 38, 40, 41, 43, 44, 46, 48) that reported dysphagia after a hemorrhagic stroke, the pooled prevalence was 58.8% (95% CI, 0.519–0.654), which was significantly higher than that after ischemic stroke, and the results were highly heterogeneous (*I*^2^ = 58.76%; *p* < 0.001).

We performed a subgroup analysis by sex (Figure 2 and Supplementary Figure 3). Of the 34 studies, 28 (15–18, 20, 22, 24–26, 28, 29, 31–33, 35–48) documented the prevalence of PSD by sex. The random-effects pooled prevalence of PSD in men was 42% (95% CI, 0.357–0.484) and in women was 46.2% (95% CI, 0.382–0.542), both with high heterogeneity (*I*^2^ = 98%; *p* < 0.001 and *I*^2^ = 97.95%; *p* < 0.001, respectively).

To investigate whether the prevalence of PSD differed between first and recurrent strokes, we performed a subgroup analysis according to the presence or absence of a stroke history (Figure 2 and Supplementary Figure 4). All 34 studies documented the prevalence of PSD after the first stroke, with a random-effects pooled prevalence of 45.1% (95% CI, 0.374–0.527) and high heterogeneity (*I*^2^ = 98.72%; *p* < 0.001). Ten of these studies (43–52) documented the prevalence of PSD in patients with a history of stroke. The random-effects pooled prevalence was 49.9% (95% CI, 0.361–0.637), with high heterogeneity. (*I*^2^ = 95.04%; *p* < 0.001).

We performed a subgroup analysis based on the time until the first dysphagia diagnosis after the stroke (Figure 2 and Supplementary Figure 5). We found that all 34 studies recruited patients in the acute phase of stroke (≤2 weeks). One study (46) evaluated within 60 days of stroke onset, but no time-specific assessment results were recorded. The remaining 33 were evaluated within 14 days of stroke onset. We stratified the prevalence of PSD in the acute phase according to the specific time of assessment. Seven studies (19, 26, 27, 43, 44, 46, 49) evaluated dysphagia within 24 h after stroke onset; the prevalence was 32.1% (95% CI, 0.272–0.370), with high heterogeneity (*I*^2^ = 85.85%; *p* < 0.001). Seven studies (20, 24, 32, 35, 41, 47, 48) evaluated dysphagia within 48 h after stroke onset; the prevalence was 45.6% (95% CI, 0.358–0.555), with high heterogeneity (*I*^2^ = 91.74%; *p* < 0.001). Seven studies (15, 17, 18, 26, 29, 30, 42) evaluated dysphagia within 72 h after stroke onset; the prevalence was 43.4% (95% CI, 0.368–0.500), with high heterogeneity (*I*^2^ = 75.36%; *p* < 0.001). In 11 studies, dysphagia was assessed within 7 days after stroke onset (15, 19, 27, 28, 31, 33, 34, 37, 38, 43, 44); the prevalence was 53.8% (95% CI, 0.399–0.676), with high heterogeneity (*I*^2^ = 99.22%; *p* < 0.001). In four studies, swallowing disorders were assessed within 7 days after stroke onset (16, 21, 25, 36). The prevalence was 48% (95% CI, 0.374–0.587), with high heterogeneity (*I*^2^ = 86.24%; *p* < 0.001).

We performed a subgroup analysis on the included studies according to the location of the stroke lesion (Figure 2 and Supplementary Figure 6). We found that most studies that documented stroke lesion sites of ischemic stroke patients reported these sites as follows and calculated the prevalence of PSD: total anterior circulation (TAC) infarct, partial anterior circulation (PAC) infarct, lacunar (LAC) infarct, and posterior circulation (POC) infarct. Due to the small number of studies on hemorrhagic stroke and the limited number of studies that documented the lesion sites, a pooled subanalysis could not be performed for hemorrhagic stroke. The results of the subgroup analysis showed that there were four studies on TAC (20, 29, 38, 47). The random-effects pooled prevalence of PSD was 64.6% (95% CI, 0.399–0.676), with high heterogeneity (*I*^2^ = 91.74%; *p* < 0.001). There were four studies on PAC (20, 29, 38, 47), with a random-effects pooled PSD prevalence of 36.2% (95% CI, 0.300–0.424); the results showed 0% heterogeneity. There were four studies (29, 38, 39, 47) on POC. The random-effects pooled prevalence of PSD was 42.4% (95% CI, 0.149–0.698), with high heterogeneity (*I*^2^ = 94.50%; *p* < 0.001). There were three studies (20, 38, 47) on LAC, and the prevalence of PSD was 44.7% (95% CI, −0.067–0.962), with high heterogeneity (*I*^2^ = 97.82%; *p* < 0.001).

We performed subgroup analyses according to the method of dysphagia diagnosis after stroke, clinical diagnosis, or instrumental diagnosis (Figure 2 and Supplementary Figure 7). Thirty studies used the clinical method to diagnose PSD (15–20, 22–24, 26, 29–48); the remaining four studies (21, 25, 27, 28) used the instrumental method. The subgroup analysis showed that the random-effects pooled prevalence of clinically assessed PSD was 43.4% (95% CI, 0.390–0.477), with high heterogeneity (*I*^2^ = 97.05%; *p* < 0.001). The random-effects pooled prevalence of instrumentally assessed PSD was 69.6% (95% CI, 0.550–0.842), and the prevalence was significantly higher than that of the clinically assessed PSD (*p* < 0.01), with high heterogeneity (*I*^2^ = 96.51%; *p* < 0.001).

We performed subgroup analyses according to the six continents covered in the study (Figure 2 and Supplementary Figure 8). Two studies (22, 30) conducted in Africa reported a PSD prevalence of 49.4% (95% CI, 0.196–0.792), with high heterogeneity (*I*^2^ = 95.7%; *p* < 0.001). Seven studies (16, 19, 23, 29, 31, 33, 37) in Asia reported a PSD prevalence of 40.1% (95% CI, 0.348–0.454), with high heterogeneity (*I*^2^ = 79.64%; *p* < 0.001). Fourteen studies in Europe (17, 22, 24, 27, 28, 32, 38–43, 45, 48) reported a PSD prevalence of 45.8% (95% CI, 0.327–0.590), with high heterogeneity (*I*^2^ = 99.42%; *p* < 0.001). The random-effects pooled prevalence of PSD based on six studies in North America (15, 20, 25, 30, 36, 44) was 44.3% (95% CI, 0.370–0.517), with high heterogeneity (*I*^2^ = 78.73%; *p* < 0.001). The random-effects pooled prevalence of PSD based on four studies in South America (34, 35, 46, 47) was 57.5% (95% CI, 0.441–0.708), with high heterogeneity (*I*^2^ = 89.93%; *p* < 0.001). The prevalence of only one study (21) in Oceania was 64.1% (95%CI, 0.558–0.724).

### 3.3 Risk factors

**Figure 4:**
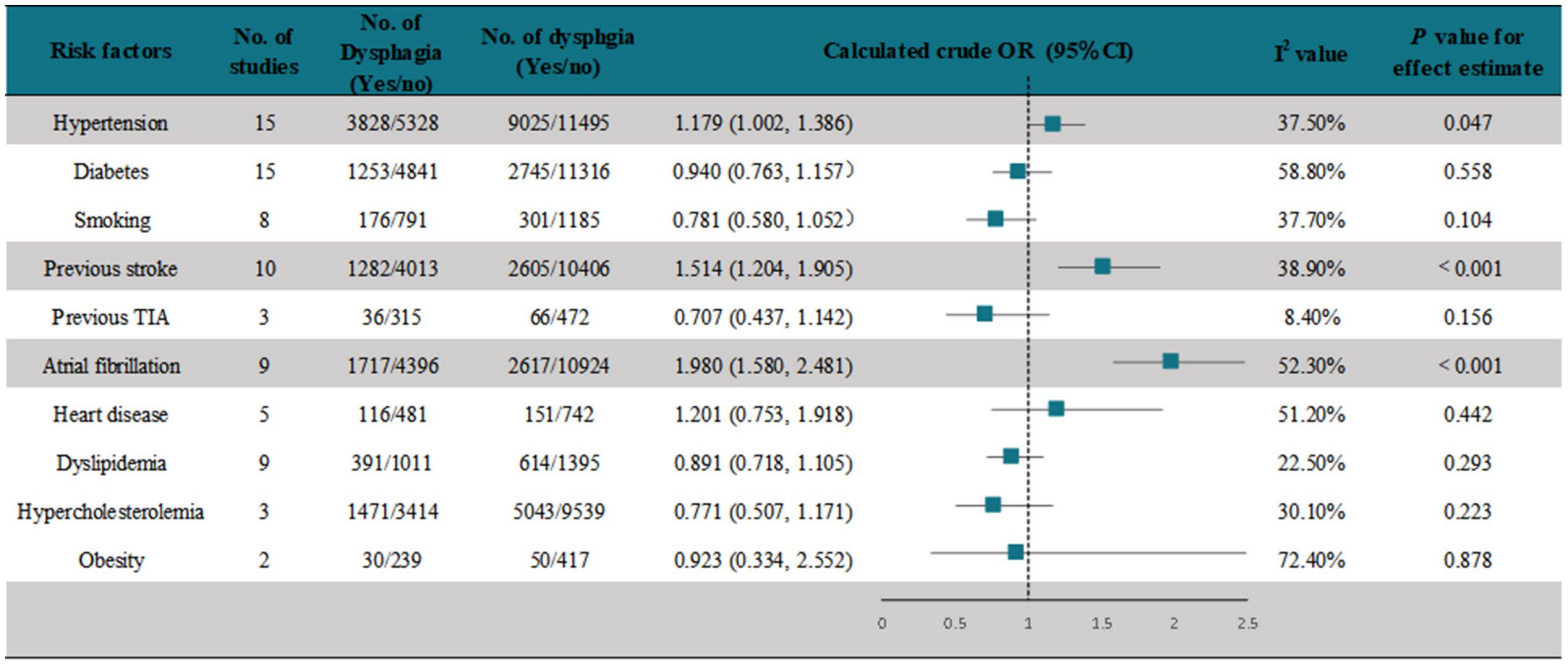
Assessment of possible risk factors for PSD.

We assessed crude ORs for risk factors of PSD documented in the studies using a random-effects model. A meta-analysis of 15 studies (22, 24, 26, 28–30, 32, 40, 42, 43, 45, 46, 49, 51) showed that hypertension was associated with the development of PSD (pooled OR = 1.179, [95% CI, 1.002–1.386], *p* < 0.05) (Figure 4 and Supplementary Figure 9). In another 15 studies (18, 20, 22, 24–26, 36, 38, 39, 41, 42, 44, 45, 47, 48) that assessed the association of diabetes with PSD, the meta-analysis showed that diabetes was not associated with the development of PSD (pooled OR = 0.940 [95% CI, 0.763–1.157], *p* < 0.05) (Figure 4 and Supplementary Figure 10). A meta-analysis of eight studies (18, 22, 25, 36, 39, 44, 45, 47) showed that smoking was not associated with the occurrence of PSD (pooled OR = 0.781, [95% CI, 0.580–1.052], *p* < 0.05) (Figure 4 and Supplementary Figure 11). A meta-analysis of 10 studies (39–48) showed that a history of stroke was significantly associated with the occurrence of PSD (pooled OR = 1.514, [95% CI, 1.204–1.905], *p* < 0.001) (Figure 4 and Supplementary Figure 12). Three studies (22, 25, 36) recorded the association of previous TIA with PSD, and the results showed no association (pooled OR = 0.707 [95% CI, 0.437–0.142], *p* > 0.05) (Figure 4 and Supplementary Figure 13). A meta-analysis of nine studies (18, 22, 24, 36, 38, 39, 42, 44, 45) showed that atrial fibrillation was significantly associated with the occurrence of PSD (pooled OR = 1.980 [95% CI, 1.580–2.481], *p* < 0.001) (Figure 4 and Supplementary Figure 14). A meta-analysis of five studies (18, 22, 41, 45, 47) showed that heart disease was not associated with the occurrence of PSD (pooled OR = 1.201 [95% CI, 0.753–1.918], *p* > 0.05) (Figure 4 and Supplementary Figure 15). A meta-analysis of nine studies (18, 20, 22, 24, 25, 36, 39, 41, 44) showed that dyslipidemia was not associated with the occurrence of PSD (pooled OR = 0.891 [95% CI, 0.718–1.105], *p >* 0.05) (Figure 4 and Supplementary Figure 16). A meta-analysis of three studies (18, 42, 44) showed that hypercholesterolemia was not associated with the occurrence of PSD (pooled OR = 0.771, [95% CI, 0.507–1.171], *p* > 0.05) (Figure 4 and Supplementary Figure 17). A meta-analysis of two studies (18, 22) showed that obesity was not associated with the occurrence of PSD (pooled OR = 0.923, [95% CI, 0.334–2.552], *p* > 0.05) (Figure 2 and Supplementary Figure 18).

In most studies, age and stroke severity were not reported as dichotomous or stratified variables, so we were unable to calculate the crude ORs; therefore, we performed a systematic descriptive analysis (Supplementary Table 3). Comparing the ages of patients with and without PSD in the included studies, whether recorded as mean or as median, PSD patients were older than those without PSD. The included studies used different scales to assess stroke severity. Fourteen studies (15, 17, 18, 20, 22, 24, 26, 28, 32, 33, 35, 42, 44, 48) used and documented in detail the National Institutes of Health Stroke Scale (NIHSS) scores of patients with and without PSD. Regardless of whether the mean or median was recorded, the NIHSS scores of patients with PSD were greater than those of PSD patients. The analysis of four studies (25, 28, 36, 48) that assessed and recorded the modified Rankin Scale (mRS) scores of patients with or without PSD in detail showed that the scores of PSD patients were higher than those of patients without PSD. The analysis of two studies (25, 36) that used and recorded the Canadian Neurological Scale (CNS) scores of patients with and without PSD showed that the CNS scores of PSD patients were lower than those of patients without PSD. Two studies (15, 28) recorded the volume of stroke lesions in patients with and without PSD, and the analysis showed that the volume of lesions in patients with PSD was significantly larger than that in PSD patients. The above analysis shows that the patient’s age and stroke severity may be risk factors for PSD. The older the patient, the more likely the patient is to develop dysphagia after stroke, and the more severe the stroke, the more likely the patient is to develop dysphagia.

### 3.4 Outcomes

**Figure 5:**
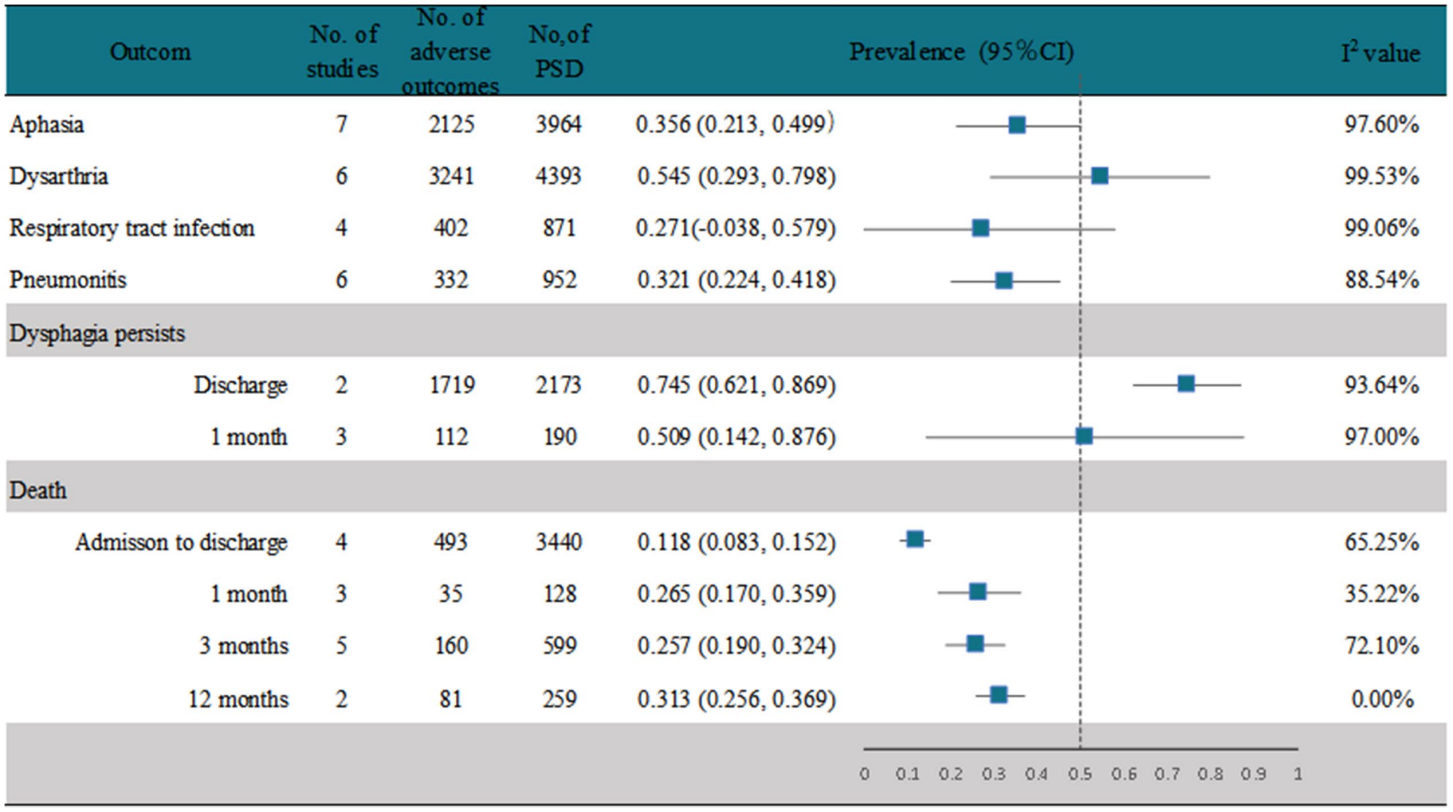
Outcome of PSD.

A random-effects analysis of seven studies (17, 28, 37, 40, 42, 47, 48) showed that the prevalence of aphasia in PSD patients was 35.6% (95% CI, 0.213–0.499), with high heterogeneity (*I*^2^ = 97.6%; *p* < 0.001). However, only 17% (95% CI, 0.07–0.27) of stroke patients without PSD had aphasia (Figure 5 and Supplementary Figure 19). Six studies (17, 28, 38, 40, 42, 47) reported the prevalence of dysarthria after PSD, and the analysis showed that 54.5% (95% CI, 0.293–0.798) of PSD patients had dysarthria, with high heterogeneity (*I*^2^ = 99.53%; *p* < 0.001), whereas 29%(95%CI, 0.17–0.41) of stroke patients without PSD had dysarthria, with high heterogeneity (*I*^2^ = 98.01%; *p* < 0.001) (Figure 5 and Supplementary Figure 20). Four studies (17, 28, 40, 41) recorded the prevalence of respiratory tract infection after PSD, and the analysis showed that 27.1% (95% CI, −0.038–0.597) of stroke patients with PSD had respiratory tract infection, with high heterogeneity (*I*^2^ = 99.06%; *p* < 0.001), whereas only 7% (95% CI, 0.01–0.12) of those without PSD had respiratory tract infection (Figure 5 and Supplementary Figure 21). Six studies (26–28, 35, 45, 48) recorded the prevalence of pneumonitis after PSD, and the analysis showed that 32.1% (95% CI, 0.224–0.418) of the stroke patients with PSD had pneumonitis, with high heterogeneity (*I*^2^ = 88.54%; *p* < 0.001). Conversely, only 7% (95% CI, 0.02–0.12) of stroke patients without PSD had pneumonitis (Figure 5 and Supplementary Figure 22). Of the studies that reported the persistence of dysphagia in PSD patients (Figure 5 and Supplementary Figure 23), 74.5% (95% CI, 0.621–0.869) in two (33, 44) of these studies had persistent dysphagia at discharge, with high heterogeneity (*I*^2^ = 93.64%; *p* < 0.001). Of these two studies, we found only one study (44) that screened for factors that still had dysphagia at discharge, and the significant correlation was NIHSS, which was higher in patients with dysphagia at discharge (*p* < 0.001). While three studies (19, 29, 43) recorded the persistence of dysphagia in patients at 1 month, the random-effects pooled prevalence was 50.9% (95% CI, 0.142–0.876), with high heterogeneity (*I*^2^ = 97.00%; *p* < 0.001). One of the studies found that dysphagia (43), which persisted up to 1 month after stroke, was significantly associated with moderate or high dependence (mRS ≥ 3, *p* < 0.001) and a BMI ≥ 20 (*p* < 0.001) as protective factors. Some studies recorded the mortality rate of PSD patients at different periods (Figure 5 and Supplementary Figure 24). Four studies (36, 39, 41, 42) recorded the mortality of PSD patients from admission to discharge, and the pooled prevalence from the random-effects model was 11.8% (95% CI, 0.083–0.152), with high heterogeneity (*I*^2^ = 65.25%; *p* = 0.03). The pooled prevalence of three studies (19, 26, 29, 43) that recorded the mortality rate of patients with PSD at 1 month was 26.5% (95% CI, 0.170–0.359), with low heterogeneity (*I*^2^ = 35.22%; *p* = 0.21). The random-effects pooled mortality prevalence of PSD patients from five studies (17, 22, 35, 41, 46) at 3 months was 25.7% (95% CI, 0.190–0.324), with high heterogeneity (*I*^2^ = 72.1%; *p* = 0.01). The random-effects pooled mortality prevalence of PSD patients recorded in two studies (39, 41) at 12 months was 31.3% (95% CI, 0.256–0.369), with no heterogeneity (*I*^2^ = 0%).

Other outcomes mentioned in the included studies were not reported as stratification variables (Supplementary Table 3), preventing any inferential analysis; therefore, we performed a systematic descriptive analysis for those variables. Nine studies (20, 28, 33, 35, 36, 40–42, 45) recorded the hospitalization period of patients with or without PSD; whether the data are expressed as mean or median, the hospitalization period of PSD patients was longer than that of patients without PSD. One study (20) used mini-nutritional assessment (MNA) to assess the nutritional state. The results showed that although patients with and without PSD were at risk of malnutrition, PSD patients had higher MNA scores than those of patients without PSD. Another study (44) also showed that the risk of malnutrition in patients without PSD was 82%, while the risk of malnutrition in PSD patients was as high as 95%. Another study (35) used the Nutritional Risk Screening tool to evaluate the nutritional status of patients, and the results showed that the probability of obtaining a Nutritional Risk Screening score of >3 (severe malnutrition) in patients with or without PSD was 23 and 14%, respectively.

### 3.5 Publication bias

We used funnel plots and Egger’s test to assess publication bias in the 34 included studies. The funnel plots showed a seemingly symmetrical distribution, while Egger’s test indicated that there was no statistically significant publication bias (*p* > 0.05; Supplementary Appendix 3). Therefore, publication bias was not the source of heterogeneity.

### 3.6 Meta-regression analysis

Because of the extremely high heterogeneity among the prevalence rates of the 34 studies and because no source of heterogeneity was found after subgroup analysis, the characteristics of the included articles, including publication year, continents, study design, and method of PSD diagnosis, were inputted as covariates in the meta-regression analysis (Supplementary Table 6). The analysis showed that publication year, continents, and study design were not the source of heterogeneity (*p* > 0.05). However, with the method of dysphagia diagnosis as a covariate, the *p*-value was <0.001, indicating that it was the main source of article heterogeneity and explaining the main reason for the inconsistencies in the prevalence of PSD.

### 3.7 Sensitivity analysis

Thirty-four studies were included in this meta-analysis. After excluding one, the combined results of the remaining studies (33) were within a 95% CI of 0.41 to 0.53, indicating stable results (Supplementary Figure 25).

## 4 Discussion

To the best of our knowledge, this is the first comprehensive meta-analysis of PSD prevalence, risk factors, and outcomes. After excluding studies with site-specific stroke, our results showed that the overall prevalence of PSD was 46.6%, which is similar to the midpoint of the prevalence range reported in previous studies (7–10). We calculated the prevalence of PSD according to the type of stroke and found that the prevalence of dysphagia after hemorrhagic stroke was higher than that after ischemic stroke (58.8% vs. 46.6%). PSD in women with stroke was more prevalent than that in men with stroke (46.2% vs. 42%). Patients with a history of stroke have a higher prevalence than those with first-time stroke (49.9% vs. 45.1%). We analyzed the location of stroke lesions and found that patients with TAC had the highest prevalence of PSD at 64.4%. Since the 34 included studies found that the study time was in the acute phase of stroke, we analyzed PSD according to the time of first diagnosis, and after excluding a study that assessed PSD within 60 days, we found that the prevalence of PSD within 24 h after stroke was not high, but gradually an upward trend was observed in 2, 3, or even 7 days, followed by a gradual decline. The prevalence of PSD was the highest in 7 days, reaching 53.8%. Overall, the prevalence of PSD in the acute phase is characterized first by an increase, a peak on day 7, and a gradual decline thereafter. Among the six continents, one study performed in Australia had the highest prevalence of PSD (64.1%), followed by four studies in South America with a prevalence of 57.9%. In the rest of the continents, the overall prevalence was between 40 and 50%. A subgroup analysis of diagnostic methods for PSD was the one that led to the largest differences in the prevalence of PSD in the current analysis. After the meta-regression analysis, the method of PSD diagnoses was the main source of heterogeneity in this study. We found that the prevalence of clinically assessed PSD was 43.4%, while the prevalence of instrumentally assessed PSD was 69.6%, which showed a statistically significant difference. This indicates that the method of diagnosis is the main reason for the inconsistency in the prevalence of PSD. The clinical evaluation methods of PSD reported in this study mainly included the Mann Assessment of Swallowing Ability, Gugging Swallowing Screen, Volume-Viscosity Swallow Test, Water Swallowing Test, the Burke dysphagia screening test (39), and Repetitive Oral Suction Swallow test (45). However, clinical assessments are always highly subjective, and most speech and language pathologists apply their clinical reasoning to customize their bedside assessments rather than using standardized assessments. Although some studies used standardized assessments, the results are mixed because the assessors were not professionals or systematically trained. Moreover, dysphagia screening protocols vary widely, and there is no consensus on the best assessment protocol (53, 54). Instrumental evaluation using video fluoroscopy remains the gold standard due to its better sensitivity and specificity. However, it requires considerable expertise and is inconvenient for routine practice, which poses great limitations for economically deprived areas and regions. Furthermore, speech therapists in different units use different food textures, doses, and sequences in the videofluoroscopy assessment process, and the evaluation and termination criteria also vary from person to person; therefore, the reliability between experts is still low (55). Research is still needed regarding the selection and application of PSD evaluation methods, and professional training of evaluators is also required to ensure that the screening of PSD is more accurate and uniform and heterogeneity is eliminated.

In this study, the possible risk factors reported in the included studies were analyzed, including hypertension, diabetes, smoking, previous stroke, previous TIA, atrial fibrillation, heart disease, dyslipidemia, hypercholesterolemia, obesity, age, and severity of stroke. Among them, hypertension was associated with the occurrence of PSD. We speculate that this result is because hypertension can cause hemorrhagic stroke, and the prevalence of PSD after hemorrhagic stroke is higher than that after ischemic stroke. Moreover, previous stroke and atrial fibrillation were significantly associated with the occurrence of PSD. Although stratified information on age and stroke severity was not available, the studies examined showed that PSD patients were older and had more severe strokes than observed in patients without PSD, so this better explains why recurrent stroke and atrial fibrillation are risk factors for PSD as they can lead to the aggravation of stroke, which makes them prone to dysphagia.

Regarding the accompanying symptoms of dysphagia, we analyzed the common complications reported in the included studies, including aphasia, dysarthria, respiratory tract infection, and pneumonitis. The most common accompanying symptoms of PSD were dysarthria, followed by aphasia, respiratory tract infection, and pneumonitis (27.1 and 32.1%, respectively). The prevalence of the above symptoms in patients with PSD was 2–4 times higher than that in patients without PSD. Our study showed that dysphagia persisted in 74.5% of the patients with PSD at discharge and in 50.9% of patients with PSD 1 month later. Moreover, patients with PSD have longer hospital stays and more severe malnutrition than that observed in patients without PSD. The mortality rate of patients with PSD reached 31.3% within 1 year and increased from admission to discharge and even to 1 month. Although the mortality rate at 3 months was slightly lower than that at 1 month, it was still high.

Although this is the first comprehensive meta-analysis of PSD prevalence, risk factors, and outcomes, shortcomings remain. First, although we found different diagnostic methods as the source of heterogeneity in PSD prevalence, we could not further classify and analyze the source of heterogeneity due to the limited number of articles. Our conclusion may not be completely plausible because of the high heterogeneity. Second, because all the included studies assessed patients in the acute phase, we did not analyze the epidemiological characteristics of PSD in the other phases, and the conclusions of this study are only applicable to the acute phase. Finally, our analysis of PSD outcomes was limited because the included studies did not have long-term follow-up cohorts.

Based on these limitations, first, there should be a more standardized and unified evaluation method for PSD and more professional evaluators involved in PSD diagnosis, as it had the greatest impact on the prevalence of PSD in this study. When assessing the prevalence of dysphagia, the method of assessment of dysphagia is important, as is the detection of aspiration, so that the importance of PSD is not underestimated. Many low-sensitivity dysphagia screens focus only on dysphagia without considering inhalation and vice versa. Dysphagia may occur in the absence of inhalation and vice versa, so using only low-sensitivity dysphagia screening has the potential to mislead results (54). Conversely, highly sensitive dysphagia screenings designed to detect aspiration and tested against FEES are more likely to depict the real situation in terms of dysphagia prevalence and risk factors (56, 57). Patients with dysphagia may have an 11-fold higher risk of developing pneumonia than non-dysphagic patients, depending on whether the dysphagia assessment method can also detect aspiration and silent aspiration (58–60). Failing to detect silent aspirators could be of particular relevance both when assessing dysphagia prevalence and for pneumonia, since a large part of pneumonia may be due to silent aspiration. In fact, stroke patients who passed low-sensitive screening for dysphagia reported higher stroke-associated pneumonia compared to those who passed high-sensitive screening, which can also detect silent aspiration (58). It can be seen that the use of some low-sensitivity assessment methods and inconsistent assessment methods will bring different results to the incidence of PSD and the occurrence and development of pneumonia after stroke. Furthermore, future studies on PSD can control for the risk factors that have a significant impact on PSD, such as hypertension, stroke history, and atrial fibrillation, develop detailed inclusion and exclusion criteria, or strictly document patients with these risk factors to ensure more accurate and reliable research conclusions and guide clinical practice more precisely. Finally, cohort studies with long-term follow-up of patients with PSD can determine their long-term outcomes and provide a better management plan in the future.

## 5 Conclusion

The overall prevalence of PSD was 46.6%, and the prevalence of dysphagia after hemorrhagic stroke was higher than that after ischemic stroke, higher in women than in men, higher in patients with a history of stroke than in patients with the first stroke, highest within 7 days of the acute phase than within other time frames, highest in patients with ischemic stroke with TAC lesions compared to the lesion sites, and highest in Australia and South America than in other continents. The prevalence of PSD was influenced most by the method of diagnosis, with the instrumental diagnosis being significantly higher than the clinical diagnosis. Hypertension, history of stroke, atrial fibrillation, patients’ age, and stroke severity were significant risk factors associated with PSD. The prevalence of aphasia, dysarthria, respiratory tract infection, and pneumonitis was 2–4 times higher in patients with PSD than in those without PSD. Our findings should be used with caution because further restrictions on the source of heterogeneity could not be made in this study. We hope that, in the future, there will be more professional personnel and a more unified evaluation method for PSD diagnosis. Furthermore, studies should be conducted with strict control or detailed documentation of risk factors, and long-term follow-up should be conducted.

## Data availability statement

The original contributions presented in the study are included in the article/Supplementary material, further inquiries can be directed to the corresponding author.

## Author contributions

WS: Conceptualization, Formal analysis, Methodology, Software, Validation, Writing – original draft. MW: Formal analysis, Methodology, Writing – review & editing. HW: Data curation, Investigation, Writing – review & editing. RP: Data curation, Investigation, Writing – review & editing. LZ: Conceptualization, Funding acquisition, Resources, Supervision, Writing – review & editing.
