# Supplementary material for: Prevalence, risk factors, and outcomes of dysphagia after stroke: a systematic review and meta-analysis
Source: Front Neurol. 2024 Jul 17;15:1403610. doi: 10.3389/fneur.2024.1403610 (PMC11288910; doi:10.3389/fneur.2024.1403610)
Supplement: Supplementary file 1 [file Table_1.docx]

**Prevalence, risk factors, and outcomes of dysphagia after stroke: A systematic review and meta-analysis**

**eTable 1. PRISMA 2020 checklist.**

| **Section and**  **Topic** | **Item #** | **Checklist item** |
| --- | --- | --- |
| **TITLE** |  |  |
| Title | **1** | Identify the report as a systematic review |
| **ABSTRACT** |  |  |
| Abstract | **2** | See the PRISMA 2020 for Abstracts checklist. |
| **INTRODUCTION** |  |  |
| Rationale | **3** | Describe the rationale for the review in the context of existing knowledge. |
| Objectives | **4** | Provide an explicit statement of the objective(s) or question(s) the review addresses |
| **METHODS** |  |  |
| Eligibility  criteria | **5** | Specify the inclusion and exclusion criteria for the review and how studies were grouped  for the syntheses. |
| Information  sources | **6** | Specify all databases, registers, websites, organisations, reference lists and other sources searched or consulted to identify studies. Specify the date when each source was last  searched or consulted. |
| Search strategy | **7** | Present the full search strategies for all databases, registers and websites, including any filters and limits used. |
| Selection  process | **8** | Specify the methods used to decide whether a study met the inclusion criteria of the review, including how many reviewers screened each record and each report retrieved,  whether they worked independently, and if applicable, details of automation tools used in the process. |
| Data collection  process | **9** | Specify the methods used to collect data from reports, including how many reviewers collected data from each report, whether they worked independently, any processes for obtaining or confirming data from study investigators, and if applicable, details of automation tools used in the process. |
| Data items | **10a** | List and define all outcomes for which data were sought. Specify whether all results that were compatible with each outcome domain in each study were sought (e.g. for all measures, time points, analyses), and if not, the methods used to decide which results to collect. |
|  | **10b** | List and define all other variables for which data were sought (e.g. participant and intervention characteristics, funding sources). Describe any assumptions made about any missing or unclear information. |
| Study risk of  bias assessment | **11** | List and define all other variables for which data were sought (e.g. participant and intervention characteristics, funding sources). Describe any assumptions made about any missing or unclear information. |
| Effect measures | **12** | Specify for each outcome the effect measure(s) (e.g. risk ratio, mean difference) used in the synthesis or presentation of results. |
| Synthesis  methods | **13a** | Describe the processes used to decide which studies were eligible for each synthesis (e.g. tabulating the study intervention characteristics and comparing against the planned groups for each synthesis (item #5)). |
|  | **13b** | Describe any methods required to prepare the data for presentation or synthesis, such as handling of missing summary statistics, or data conversions. |
|  | **13c** | Describe any methods used to tabulate or visually display results of individual studies and syntheses. |
|  | **13d** | Describe any methods used to synthesize results and provide a rationale for the choice(s). If meta-analysis was performed, describe the model(s), method(s) to identify the presence and extent of statistical heterogeneity, and software package(s) used. |
|  | **13e** | Describe any methods used to explore possible causes of heterogeneity among study results (e.g. subgroup analysis, meta-regression). |
|  | **13f** | Describe any sensitivity analyses conducted to assess robustness of the synthesized results. |
| **Section and**  **Topic**  Reporting bias  assessment | **Item #**  **14** | **Checklist item** |
|  |  | Describe any methods used to assess risk of bias due to missing results in a synthesis  (arising from reporting biases). |
| Certainty  assessment | **15** | Describe any methods used to assess certainty (or confidence) in the body of evidence for  an outcome. |
| **RESULTS** |  |  |
| Study selection | **16a** | Describe the results of the search and selection process, from the number of records identified in the search to the number of studies included in the review, ideally using a flow diagram. |
|  | **16b** | Cite studies that might appear to meet the inclusion criteria, but which were excluded,  and explain why they were excluded |
| Study  characteristics | **17** | Cite each included study and present its characteristics. |
| Risk of bias in  studies | **18** | Present assessments of risk of bias for each included study. |
| Results of  individual  studies | **19** | For all outcomes, present, for each study: (a) summary statistics for each group (where  appropriate) and (b) an effect estimate and its precision (e.g. confidence/credible  interval), ideally using structured tables or plots. |
| Results of  syntheses | **20a** | For each synthesis, briefly summarise the characteristics and risk of bias among  contributing studies. |
|  | **20b** | Present results of all statistical syntheses conducted. If meta-analysis was done, present  for each the summary estimate and its precision (e.g. confidence/credible interval) and  measures of statistical heterogeneity. If comparing groups, describe the direction of the  effect. |
|  | **20c** | Present results of all investigations of possible causes of heterogeneity among study results |
|  | **20d** | Present results of all sensitivity analyses conducted to assess the robustness of the synthesized results. |
| Reporting  biases | **21** | Present assessments of risk of bias due to missing results (arising from reporting biases)  for each synthesis assessed. |
| Certainty of  evidence | **22** | Present assessments of certainty (or confidence) in the body of evidence for each  outcome assessed. |
| **DISCUSSION** |  |  |
| Discussion | **23a** | Provide a general interpretation of the results in the context of other evidence |
|  | **23b** | Discuss any limitations of the evidence included in the review. |
|  | **23c** | Discuss any limitations of the review processes used. |
|  | **24c** | Discuss implications of the results for practice, policy, and future research |
| **OTHER INFORMATION** |  |  |
| Registration  and protocol | **24a** | Provide registration information for the review, including register name and registration  number, or state that the review was not registered. |
|  | **24b** | Indicate where the review protocol can be accessed, or state that a protocol was not  prepared |
|  | **24c** | Describe and explain any amendments to information provided at registration or in the  protocol. |
| Support | **25** | Describe sources of financial or non-financial support for the review, and the role of the  funders or sponsors in the review |
| Competing  interests | **26** | Declare any competing interests of review authors. |
| Availability of  data, code and  other materials | **27** | Report which of the following are publicly available and where they can be found:  template data collection forms; data extracted from included studies; data used for all  analyses; analytic code; any other materials used in the review. |

**eTable 2. MOOSE checklist.**

| **Section/Topic** | **MOOSE checklist Item(s)** |
| --- | --- |
| **BACKGROUND** |  |
| Reporting of  background should  include | Problem definition. Hypothesis statement. Description of study outcome(s). Type of exposure or intervention used. Type of study designs used. Study population. |
| **SEARCH** |  |
| Reporting of search  strategy should  include | Qualifications of searchers (eg, librarians and investigators). Search strategy, including time  period included in the synthesis and keywords. Effort to include all available studies, including contact with authors. Databases and registries searched. Search software used, name and version, including special features used (eg, explosion). Use of hand searching (eg, reference lists of obtained articles). List of citations located and those excluded, including justification. Method of addressing articles published in languages other than English. Method of handling abstracts and unpublished studies.Description of any contact with authors. |
| **METHODS** |  |
| Reporting of methods  should include | Description of relevance or appropriateness of studies assembled for assessing the hypothesis to be tested. Rationale for the selection and coding of data (eg, sound clinical principles or convenience). Documentation of how data were classified and coded (eg, multiple raters, blinding, and interrater reliability). Assessment of confounding (eg, comparability of cases and controls in studies where appropriate). Assessment of study quality, including blinding of quality assessors; stratification or regression on possible predictors of study results. Assessment of heterogeneity. Description of statistical methods (eg, complete description of fixed or random effects models, justification of whether the chosen models account for predictors of study results, dose-response models, or cumulative meta-analysis) in sufficient detail to be replicated. Provision of appropriate tables and graphics. |
| **RESULTS** |  |
| Reporting of results  should include | Graphic summarizing individual study estimates and overall estimate. Table giving descriptive information for each study included. Results of sensitivity testing (eg, subgroup analysis).  Indication of statistical uncertainty of findings. |
| **DISCUSSION** |  |
| Reporting of  discussion should  include | Quantitative assessment of bias (eg, publication bias). Justification for exclusion (eg, exclusion of non–English-language citations). Assessment of quality of included studies. |
| **CONCLUSIONS** |  |
| Reporting of  conclusions should  include | Consideration of alternative explanations for observed results. Generalization of the  conclusions (ie, appropriate for the data presented and within the domain of the literature review). Guidelines for future research. Disclosure of funding source. |

**Appendix 1. Literature search strategy**

**1.MEDLINE(via Web of Science)**

1. #2:((((((((((((((((((((((((((((TS=(stroke)) OR TS=(Cerebrovascular Accident)) OR TS=(Cerebrovascular Accidents)) OR TS=(CVA (Cerebrovascular Accident) )) OR TS=( (Cerebrovascular Accident))) OR TS=(Cerebrovascular Apoplexy)) OR TS=(Apoplexy, Cerebrovascular)) OR TS=(Vascular Accident, Brain)) OR TS=(Brain Vascular Accident)) OR TS=(Brain Vascular Accidents)) OR TS=(Vascular Accidents, Brain)) OR TS=(Cerebrovascular Stroke)) OR TS=(Cerebrovascular Strokes)) OR TS=(Stroke, Cerebrovascular)) OR TS=(Strokes, Cerebrovascular)) OR TS=(Apoplexy)) OR TS=(Cerebral Stroke)) OR TS=(Cerebral Strokes)) OR TS=(Stroke, Cerebral)) OR TS=(Strokes, Cerebral)) OR TS=(Stroke, Acute)) OR TS=(Acute Stroke)) OR TS=(Acute Strokes)) OR TS=(Strokes, Acute)) OR TS=(Cerebrovascular Accident, Acute)) OR TS=(Acute Cerebrovascular Accident)) OR TS=(Acute Cerebrovascular Accidents)) OR TS=(Cerebrovascular Accidents, Acute))

2.#3: (((((((((TS=(dysphagia)) OR TS=(Deglutition Disorder)) OR TS=(Disorders, Deglutition)) OR TS=(Swallowing Disorders)) OR TS=(Swallowing Disorder)) OR TS=(Oropharyngeal Dysphagia)) OR TS=(Dysphagia, Oropharyngeal)) OR TS=(Esophageal Dysphagia)) OR TS=(Dysphagia, Esophageal)) OR TS=(swallowing dysfunction)

3. (#2 AND #3) AND (PY==("2021" OR "2020" OR "2019" OR "2018" OR "2017" OR "2016" OR "2015" OR "2014" OR "2013" OR "2012" OR "2011" OR "2010" OR "2009" OR "2008" OR "2007" OR "2006" OR "2005" OR "2004" OR "2003" OR "2002" OR "2001" OR "2000" OR "1999" OR "1998" OR "1997" OR "1996" OR "1995" OR "1994" OR "1993" OR "1992" OR "1991" OR "1990" OR "1989" OR "1988" OR "1987" OR "1986" OR "1985" OR "1984" OR "1983" OR "1982" OR "1981" OR "1979" OR "1975" OR "1973" OR "1969" OR "1967" OR "1956"))

**2.Pubmed**：

Search: (("Stroke"[MeSH Terms] OR ("Strokes"[Title/Abstract] OR "cerebrovascular accident"[Title/Abstract] OR "cerebrovascular accidents"[Title/Abstract] OR (("Stroke"[MeSH Terms] OR "Stroke"[All Fields] OR "cva"[All Fields]) AND "cerebrovascular accident"[Title/Abstract]) OR ("CVAs"[All Fields] AND "cerebrovascular accident"[Title/Abstract]) OR "cerebrovascular apoplexy"[Title/Abstract] OR "apoplexy cerebrovascular"[Title/Abstract] OR "vascular accident brain"[Title/Abstract] OR "brain vascular accident"[Title/Abstract] OR "brain vascular accidents"[Title/Abstract] OR (("blood vessels"[MeSH Terms] OR ("blood"[All Fields] AND "vessels"[All Fields]) OR "blood vessels"[All Fields] OR "Vascular"[All Fields] OR "neovascularization, pathologic"[MeSH Terms] OR ("neovascularization"[All Fields] AND "pathologic"[All Fields]) OR "pathologic neovascularization"[All Fields] OR "vascularisation"[All Fields] OR "vascularization"[All Fields] OR "vascularisations"[All Fields] OR "vascularise"[All Fields] OR "vascularised"[All Fields] OR "vascularities"[All Fields] OR "vascularitis"[All Fields] OR "vascularity"[All Fields] OR "vascularizations"[All Fields] OR "vascularize"[All Fields] OR "vascularized"[All Fields] OR "vascularizes"[All Fields] OR "vascularizing"[All Fields] OR "vasculars"[All Fields]) AND "accidents brain"[Title/Abstract]) OR "cerebrovascular stroke"[Title/Abstract] OR "cerebrovascular strokes"[Title/Abstract] OR "stroke cerebrovascular"[Title/Abstract] OR "strokes cerebrovascular"[Title/Abstract] OR "Apoplexy"[Title/Abstract] OR "cerebral stroke"[Title/Abstract] OR "cerebral strokes"[Title/Abstract] OR "stroke cerebral"[Title/Abstract] OR "strokes cerebral"[Title/Abstract] OR "stroke acute"[Title/Abstract] OR "acute stroke"[Title/Abstract] OR "acute strokes"[Title/Abstract] OR "strokes acute"[Title/Abstract] OR "cerebrovascular accident acute"[Title/Abstract] OR "acute cerebrovascular accident"[Title/Abstract] OR "acute cerebrovascular accidents"[Title/Abstract] OR "cerebrovascular accidents acute"[Title/Abstract])) AND ("Deglutition Disorders"[MeSH Terms] OR ("deglutition disorder"[Title/Abstract] OR "disorders deglutition"[Title/Abstract] OR "swallowing disorders"[Title/Abstract] OR "swallowing disorder"[Title/Abstract] OR "Dysphagia"[Title/Abstract] OR "oropharyngeal dysphagia"[Title/Abstract] OR "dysphagia oropharyngeal"[Title/Abstract] OR "esophageal dysphagia"[Title/Abstract] OR "dysphagia esophageal"[Title/Abstract] OR "swallowing dysfunction"[Title/Abstract]))) AND (1955:2021[pdat])

**3. Web of science：**

1.#45:((((((((((((((((((((((((((((TS=(stroke)) OR TS=(Cerebrovascular Accident)) OR TS=(Cerebrovascular Accidents)) OR TS=(CVA (Cerebrovascular Accident) )) OR TS=( (Cerebrovascular Accident))) OR TS=(Cerebrovascular Apoplexy)) OR TS=(Apoplexy, Cerebrovascular)) OR TS=(Vascular Accident, Brain)) OR TS=(Brain Vascular Accident)) OR TS=(Brain Vascular Accidents)) OR TS=(Vascular Accidents, Brain)) OR TS=(Cerebrovascular Stroke)) OR TS=(Cerebrovascular Strokes)) OR TS=(Stroke, Cerebrovascular)) OR TS=(Strokes, Cerebrovascular)) OR TS=(Apoplexy)) OR TS=(Cerebral Stroke)) OR TS=(Cerebral Strokes)) OR TS=(Stroke, Cerebral)) OR TS=(Strokes, Cerebral)) OR TS=(Stroke, Acute)) OR TS=(Acute Stroke)) OR TS=(Acute Strokes)) OR TS=(Strokes, Acute)) OR TS=(Cerebrovascular Accident, Acute)) OR TS=(Acute Cerebrovascular Accident)) OR TS=(Acute Cerebrovascular Accidents)) OR TS=(Cerebrovascular Accidents, Acute))

2.#46:(((((((((TS=(dysphagia)) OR TS=(Deglutition Disorder)) OR TS=(Disorders, Deglutition)) OR TS=(Swallowing Disorders)) OR TS=(Swallowing Disorder)) OR TS=(Oropharyngeal Dysphagia)) OR TS=(Dysphagia, Oropharyngeal)) OR TS=(Esophageal Dysphagia)) OR TS=(Dysphagia, Esophageal)) OR TS=(swallowing dysfunction)

3.(#45 AND #46) AND (PY==("2021" OR "2020" OR "2019" OR "2018" OR "2017" OR "2016" OR "2015" OR "2014" OR "2013" OR "2012" OR "2011" OR "2010" OR "2009" OR "2008" OR "2007" OR "2006" OR "2005" OR "2004" OR "2003" OR "2002" OR "2001" OR "2000" OR "1999" OR "1998" OR "1997" OR "1996" OR "1995" OR "1994" OR "1993" OR "1992" OR "1991" OR "1990" OR "1989" OR "1988" OR "1987" OR "1986" OR "1985"))

**4. Cochrane Library:**

(stroke) OR (Cerebrovascular Accident) OR (Cerebrovascular Accidents)OR(CVA)OR (Cerebrovascular Accident) OR (Cerebrovascular Apoplexy) OR (Apoplexy, Cerebrovascular) OR (Vascular Accident, Brain) OR (Brain Vascular Accident)OR (Brain Vascular Accidents) OR (Vascular Accidents, Brain) OR (Cerebrovascular Stroke) OR (Cerebrovascular Strokes) OR (Stroke, Cerebrovascular) OR (Strokes, Cerebrovascular) OR (Apoplexy) OR (Cerebral Stroke) OR (Cerebral Strokes) OR (Stroke, Cerebral) OR (Strokes, Cerebral) OR (Stroke, Acute) OR (Acute Stroke) OR (Acute Strokes) OR (Strokes, Acute) OR (Cerebrovascular Accident, Acute) OR (Acute Cerebrovascular Accident) OR (Acute Cerebrovascular Accidents) OR (Cerebrovascular Accidents, Acute) in Title Abstract Keyword AND (dysphagia) OR (Deglutition Disorder) OR (Disorders, Deglutition) OR (Swallowing Disorders) OR (Swallowing Disorder) OR (Oropharyngeal Dysphagia) OR (Dysphagia, Oropharyngeal) OR (Esophageal Dysphagia) OR (Dysphagia, Esophageal) OR (swallowing dysfunction) in Title Abstract Keyword - with Cochrane Library publication date to Dec 2021 (Word variations have been searched)

**5. Embase:**

('cerebrovascular accident'/exp OR stroke:ti,ab,kw OR strokes:ti,ab,kw OR 'cerebrovascular accidents':ti,ab,kw OR 'cerebrovascular accident':ti,ab,kw OR 'cerebrovascular apoplexy':ti,ab,kw OR 'apoplexy, cerebrovascular':ti,ab,kw OR 'vascular accident, brain':ti,ab,kw OR 'brain vascular accident':ti,ab,kw OR 'brain vascular accidents':ti,ab,kw OR 'cerebrovascular stroke':ti,ab,kw OR 'cerebral stroke':ti,ab,kw OR apoplexy:ti,ab,kw OR 'acute stroke':ti,ab,kw OR 'strokes, acute':ti,ab,kw OR 'cerebrovascular accident, acute':ti,ab,kw OR 'acute cerebrovascular accident':ti,ab,kw OR 'acute cerebrovascular accidents':ti,ab,kw) AND ('dysphagia'/exp OR 'deglutition difficulty':ti,ab,kw OR 'deglutition disorder':ti,ab,kw OR 'deglutition disorders':ti,ab,kw OR 'difficult deglutition':ti,ab,kw OR 'difficulty in swallowing':ti,ab,kw OR 'difficulty swallowing':ti,ab,kw OR dysphagias:ti,ab,kw OR 'swallowing difficult':ti,ab,kw OR 'swallowing difficultness':ti,ab,kw OR 'swallowing difficulty':ti,ab,kw OR 'swallowing disorder':ti,ab,kw OR 'swallowing dysfunction':ti,ab,kw OR 'oropharyngeal dysphagia':ti,ab,kw OR 'esophageal dysphagia':ti,ab,kw) AND [1967-2021]/py

eTable 3. Characteristics of the included literature

| **Author&**  **Year** | **Country Continent** | **Study time (follow up, y)** | **Age, y** | **Type of stroke/ Lesion side,n &duration** | **Stroke severity assessment**  **(test score, n)** | **PSD,n** | **Total,n** | **Diagnostic Method &time(d) of PSD** | **Severity of PSD** | **Risk factor** | **outcomes,n** |
| --- | --- | --- | --- | --- | --- | --- | --- | --- | --- | --- | --- |
| MG  2008  [15] | *Baltimore*  North America | 2001-2005 | D: 62.6 ±14.3  N: 57.2 ±19.5 | ischemic stroke  (within 24 h) | NIHSS score  D: 11.4±5.3  N: 6.2 ±5.9  volume, cc  D: 51.7 ±44.9  N: 23.0 ±30.6 | 14  M:7  F:7 | 29  M:14  F:15 | BSA  ＜24h  D:9 N:8  ≤3days  D:10 N:10  ≤7days  D:13 N:11  ＞7days  D:1 N:1 | N/A | Black/white  N:6/8 N:8/6 | N/A |
| Zara  2021  [16] | *Iran*  Asia | 2018.2-2019.2 | 62.61±14.89  N:61.22 ±13.7 D:65.14 ±16.6 | Ischemic,93  Hemorrhagic,7  (6.17 ± 3.43d) | MMSE test score  Mild  5.28 ± 3.59,25  Moderate  15.90 ± 3.03,36  Severe  22.64 ± 1.26,21  Without  28.25 ± 2.01,18 | 36  M:22  F:14 | 100  M:56  F:44 | MASA  (6.17 ± 3.43) | RHD:  125 ±40  LHD:  114 ±44 | N/A | N/A |
| Antía  2019  [17] | *Spain*  Europe | 2017.8-2018.4  (3month follow-up) | 72.05 ± 13.47  D:76.40±11.50  N:66.37±13.85 | Ischaemic,86 D:48 N:38  Hemorrhagic,20 D:12 N:8  (acute) | NIHSS score  5.49 ± 5.44  D: 6.81 ± 5.83  N: 3.38 ± 3.46 | 60  M:29  F:31 | 106  M:58  F:48 | V-VST  (acute, Within 72 hours of admission) | N/A | N/A | Admission  Aphasia  D:9 N:7  Dysarthria  D:26 N:18  3 monthes follow-uo  Respiratory tract infection:D:12N:3  Death (n:12)  - Due to respiratory tract infection (D:8 N:1)  - Due to other causes (D:4 N:1) |
| Eman  2021  [18] | *Egyp*t  Africa | 2015.10-2016.3 | 54.6 ± 11.3  Ischemic:  D:58.3 ± 8.55 N: 55.2 ± 10.6  Hemorrhagics:  D: 51.1 ± 13.4  N: 49.8 ± 13.3 | Ischemic,180 D:57 N:123  Hemorrhagics,70 D:41 N:29  (within 72 h of onset) | NIHSS score  Ischemic：  D:13.6 ± 6.1  N: 7.3 ± 3.6  Hemorrhagics  D:13.8±6  N:6.1±2.5 | 98  M:48  F:50 | 250  M:122  F:128 | WST  (within 72 h of onset) | DOSS  Ischemic/  hemorrhagics:  Mild:  15 /10  Moderate:  21/17  Severe  21/14 | Ischemic/hemorrhagics:  Hypertension,  D: 11/27 N:41/19  Diabetes mellitus  D:24/ 13 N:29/10  Smoking D:12/4 N:29/6  Obesity D:13/10 N:20/6  Dyslipidemia  D:10/8  N:14/5  Substance abuse  D:4/8 N:6/5  Ischemic heart Disease  D:7/1 N: 8/2  Hyperuricemia  D:2/2 N:11/3  Atrial fibrillation  D:14/0 N:5 /1 | N/A |
| Hamidon  2006  [19] | *Malaysia*  Asia | 2004.7-2004.12  (1month follow-up) | 64.4 ± 10.9  D: 67.5 ± 11.1 N:62.3 ± 10.3 | Ischaemic  前循环  Middle cerebral artery,53  Anterior cerebral artery,8  后循环  Posterior cerebral artery,10  Lacunar infarct,60  (acute) | N/A | 55 | 134  M:67  F:67 | BSA  (3-7d) | N/A | Age> 75*  Diabetes Mellitus*  Hypertension  MCA infarct*  (within 1 month) | persistent dysphagia at 1 month, 29  Death, 14 |
| Michael  2012  [20] | *America*  North America | N/A | 65.7  D: 65.6±13.0  N: 65.5±13.4 | Ischaemic  total anterior circulation  infarct, 12 D:11 N:1  partial anterior circulation infarct,30 D:12 N:18  lacunar infarct,18 D:2 N:16  posterior circulation infarct,7  D:0 N:7 (1.4d） | NIHSS Score  9.18±7.29  D:15.84±5.78  N:4.93±4.47 | 25  M:10  F:15 | 67  M:29  F:38 | MASA  (1.4d) | MASA:  D:125.6 ±42.89  N:194.1 ±5.62  FOIS:  D: 2.84 ±2.08  N: 2.84 ±2.08 | Caucasian  D:15 N:14  African American  D:9 N:27  Diabetes mellitus  D:7 N: 12  Hyperlipidemia  D:7 N:9  Hypertension  D:17 N:29 | mean length of hospital stay:  D:4.92±2.82  N:2.39±1.87  Barthel index  D: 15.40 ±23.06  N: 77.63 ±23.15  mini nutritional assessment（MNA）  D:22.94 ±4.09  N: 23.38 ±3.75 |
| Giselle  2000  [21] | Australia  Oceania | 1994.5-1995.5 | 71±12.1 | Ischemic,117  Hemorrhagics,10  (＜7 days since symptom  onset) | N/A | 82 | 128  M:82  F:46 | VFSS  withing 10 d(0-47)  Clinical assessment | Mild,37moderate ,39  Severe, 6 | N/A | N/A |
| Maurizio  2004  [22] | *Italy*  Euorpe | 2001.4-  2002.12  (3 month follow-up) | 73.2±11.4  D:76.2 ±10.8  N:71.5±11.9 | Ischemic,343 D:110  Hemorrhagics,63 D:31  (Acute) | NIHSS Score  ≥15,  T:223 D:85 N:9 | 141  M:68  F:73 | 406  M:222  F:184 | BSA  （5.5h）  (1-12h) | N/A | Hypertension，  D:78 N:166  Diabetes mellitus  D:24 N:57  Smoking D:24 N:84  Hyperlipidemia  D: 23 N:76  Ischemic heart disease  D:26 N:62  TIA D: 5 N:24  Atrial fibrillation  D:35 N:36  Alcoholism D:3 N:11  Obesity D:7 N:24  Somnolence D:55 N:3 | Persistent dysphagia at 3 month,2  Death,D:55 N:9  recurrence of stroke,  D: 16 N:8 |
| Mahsa  2021[23] | *Iran*  Asia | 2020.4-2020.11 | 70.91 ± 11.07 | Ischemic,327  Hemorrhagics,22  (48h) | NIHSS Score | 136 | 349  M:186  F:163 | NDPCS  within 12 to 24 h after admission | N/A | N/A | N/A |
| Anna  2017[24] | *Italy*  Europe | 2013.2-  2014.7  (1 month follow-up) | 66.9± 11.9  D: 68.2 ± 11.2  N: 65.1 ± 12.7 | Ischemic,  (＜72h) | NIHSS Score  (9.0±7.1)  D:12.2 ± 7.5  N:4.7 ± 2.9 | 81  M:43  F:38 | 140  M:76  F:64 | GUSS  (＜48h following stroke presentation) | 12.5±7.8 | Hypertension  D:409 N:35  Diabetes D: 20 N:19  Atrial ﬁbrillation  D:22 N:18  Dyslipidemia  D:36 N:26  Obstructive sleep apnea D:54 N: 18 | N/A |
| Heather  2017[25] | *Canada*  North America | N/A | 66.7±15  D:69.9±13.8  N:63.6±15.6 | Ischemic  (within 14 days of stroke onset） | CNS（8±5）  D:6.7±2.7  N:9.2±2.3  Mean Rankin scale score  D:3.6±1.2  N:2.3±1.4 | 76  M:46  F:30 | 160  M:91  F:69 | instrumental assessment  （within 14 days） | N/A | Hypertension  D:59 N:52  Diabetes，D: 22 N:16  Hyperlipidemia  D: 26 N:29  Previous TIA  D: 14 N:17  Current smoker  D: 15 N:18 | N/A |
| Sani  2017[26] | *Nigeria*  Africa | 2015.5-2017.1 | 55.51±15.7  D:55.8±16.6  N:55.3±15.3 | both ischmic and haemorrhagic  (within 72 h of onset of symptoms) | NIHSS Score  (9.47±6.3)  D: 13.28±6.9  N: 7.50±5.1 | 32  M:17  F:15 | 94  M:53  F:41 | WST  (72h) | N/A | High blood pressure,  D: 30 N:57  Diabetes mellitus,  D: 4 N:12  Sickle cell disease,  D: 0 N:1  Cardiac diseases,  D: 0 N:3 | 1 month：  Death,D: 6 N:7  Aspiration pneumonitis  D:10 N:2  Handicapped  D:13 N:14 |
| Suntrup-Krueger  2017[27] | *Germany*  Europe | 2008.10-2010.3 | 73.7±12.2 | Ischaemic/haemorrhagic  160/40  (Acute) | NIHSS Score  9.7±5.2 | 165 | 200  M:101  F:99 | VFSS  (within 96 h from admission) | N/A | N/A | Pneumonia，D:48 N:2 |
| Bendix  2018[28] | *Germany*  Europe | 2004-2014 | 71.01±12.8  D: 71.4±12.9  N:69.3±12.1 | Ischaemic D:513 N:103  Haemorrhagic D:58 N:13  (＜96h)  Supratentorial D:470 N:96  Infratentorial D:86 N:20  Multiple locations D:15 N:0 | NIHSS Score  11.2±6.2  D:11.8±6.2  N:8.2±5.1  mRS  3.8±1.3  D: 4.0±1.2  N: 3.1±1.4 | 571  M:309  F:262 | 687  M: 375  F:312 | FEES  (within 96 h from post stroke) | FEEDS  4.1±1.3  FEDSS 1 N:116  FEDSS 2, 74  FEDSS 3, 147  FEDSS 4, 122  FEDSS 5, 125  FEDSS 6, 103 | Hypertension  D:43 N:9 | Hemiparesis  D:520 N:93  Facial paralysis  D:482 N:87  Dysarthria D:323 N:58  Aphasia, D:231 N:47  Neglect ,D:160 N:15  Need for intubation  D:195 N:19  Tracheotomy D:120N:3  InfectionsD:361 N:29  PneumoniaD:207N:11  Total length of stay in hospital,D:17.68±13.72 N:13.81±9.66 |
| Zeki  2010[29] | Iraqi  Asia | 2007.7-2008.2 | 60.55±11.81  30-40 D:2 N:4  41-50 D:4 N:4  51-60 D:11 N:8  61-70 D:12 N:8  71-82 D:12 N:7 | Ischaemic D:38 N:30  Haemorrhagic D:3 N:1  total Anterior circulation stroke (TACS) D:10 N:0  partial Anterior circulation stroke (PACS) D:18 N:23  Posterior circulation stroke (PCS):  lateral medullary syndrome(LMS) D:10 N:1  and posterior cerebral artery (PCA) stroke.D: 3 N:7 | mRS  Mild(0-2):  D:10 N:21  Medorately severity(3-5):  D:31 N:10 | 41  M:21  F:20 | 72  M:40  F:32 | MASA  (72 hours） | MASA  Mild 168-177  n=20  Moderate  139-167  n=6  Severe≤138  n=15 | N/A | 1 month follow-up(22)  Persistent dysphagia  D:8  Death:15 |
| Kaila  2019[30] | *Boston*  North America | N/A | 72.33±14.40 | Ischemic  Brainstem 10 | N/A | 32 | 100  M:63  F:37 | (clinical swallow evaluation）  Within 72h | N/A | Hypertension 58  Hyperlipidemia 21  Heartdisease/myocardial infarction 19  Diabetes 15  Cancer 11  Atrial fibrillation 9 | N/A |
| Shiva  2019  [31] | *Iran*  Asia | N/A | 62.82±15.54  D:71.62±12.90  N:58.50±14.97 | Ischaemic,72 D:24,N:48  Haemorrhagic,14 D:5.N:9  Subarachnoid Hemorrhage,2  D:0,N:2  (3.5±2.7) | MoCA Score  D: 9.65±5.16  N: 15.27±7.93 | 29  M;14  F:15 | 88  M:54  F:34 | MASA  Mild:168–  177 ,n=10, Moderate 139-167,  n=12,  Severe<138  n=7 | N:192.47±  6.30 D:152.24±21.64  (3.5±2.7) | N/A | N/A |
| Elien  2020[32] | *Belgium*  Europe | 2018.3-2019.10 | 67±14  D:72±15  N:66±13 | Ischemic  Infratentorial D:3 N:12  Mixed D:2 N:10  （within 48 hours ） | NIHSS score  D:14(5-18)  N: 3(1-7)  (N/A) | 35  M:19  F:16 | 151  M:85  F:66 | MASA  (within 48 hours) | N/A | N/A | N/A |
| Nayeon  2021[33] | *Korea*  Asia | 2012.8-2015.5 | 66.78±12.9  D: 67.3±11.0  N: 64.1±12.3 | Ischemic,  Cortex,D:924 N:538  Subcortex,D:855 N:1673  Brainstem,D:379 N:636  Cerebellum,D:135 N:379 | NIHSS score  （4.8±5.3）  D: 7.9±6.5  N: 3.2±3.5 | 1940  M:1045  F:895 | 5740  M:4347  F:1393 | WST  (at post-stroke 7ds) | ASHA-NOMS  Level1/2/3/4/5/6/7  607/46/59/105/322/801/0 | N/A | Days at hospital,  D:21.5±18.8  N: 11.3±11.1  Dysphagia at discharge,  1561(n=33mising data) |
| Danielle  2016[34] | South America | N/A | 65.7 ± 14.4 | Ischemic,39 D:29 N:10  Hemorrhagic,1 D:1 N:0  Ischemic with Hemorrhagic transformation,2 D:2 N:0  （within 48 hours） | NIHSS score  Minor (0-6),17  Moderate  (7-15),16  Severe (≥16),9 | 32 | 42  M:20  F:22 | PIODA.  4 ± 1.91  (within 7 dyas） | GUSS  Normal, 10  Mild, 5  Moderate,10  Severe,17 | N/A | N/A |
| Juli  2019[35] | *Brazil*  South  America | 2014.4-  2017.11 | Median:  D:70.5(62-79)  N:67(54-75) | Ischemic D:77 N:109  Hemorrhagic D:9 N:6  (＜72h＝ | NIHSS score  D: 10 (5-16)  N: 3 (1-6) | 86  M:44  F:42 | 201  M:110  F:91 | V-VST  (ﬁrst 48 h after  Admission) | FOIS | N/A | Length of stay  N: 6 (4-8) D:7 (4-10.2)  Pneumonia D:17 N:3  Nutritional Risk Screening＞3  D: 20 N:17  3 months follow-up：  mRS＞3 D:46 N:12  Mortality D:17 N:1 |
| Heather  2013[36] | *Canada*  North America | 2003.7-2008.3 | 68±15  D:71±14  N:65±16 | Ischemin  （median 75h(108h)  within 2 weeks） | CNS score  8.1±3  D:6.5±2.8  N:9.2±2.5  mRS  D:3.7±1.2  N:2.1±1.4 | 98  M:58  F:40 | 221  M:123  F:98 | speech-language pathologist clinical assessment  （within 2 weeks） | N/A | Hypertension  D:74 N:80  Diabetes  D:27 N:33  Hyperlipidemia  D:31N:43  Previous TIA  D:17 N:25  Atrial Fibrillation  D:11 N:9  Current smoker  D: 23 N:24  Asthma or COPD  D:7 N:8  Cancer  D:15 N:8  Dementia  D:7 N:5 | length of stay (median,range)  D:14（0-221）  N:7（0-62）  Discharge dead  D:9 N:＜5 |
| Shiva  2016[37] | *Iran*  Asia | 2014.4-  2014.9 | 64±37  D:71.6±12.44  N:58.24±14.76 | Ischemic,D:8 N:50  Hemorrhagic,D: 12 N:9  （24-72h） | N/A | 54  M:24  F:30 | 113  M:69  F:44 | MASA  3.8 ±2.9 | D:139.61±29.96  N:192.71±6.32 | N/A | Aphasia  D: 8  N:1 |
| Smthards  2007[38] | *London*  Europe | 1995-1998 | D: 74.3 ±13.0  N: 69.6 ±14.0 | Ischemic  posterior cerebral infarction (POCI), D:89 N:58  total anterior cerebral infarction (TACI),  D:89 N:163  partial anterior cerebral infarction (PACI),  D:47 N:94  lacunar infarction (LACI),  D: 152 N:31  primary intracerebral haemorrhage (PICH),  D: 29 N:29  subarachnoid haemorrhage (SAH) D:88 N:32  unclassiﬁed D:399 N:320 | GCS  MRC  (N/A） | 567  M:247  F:320 | 1188  M:571  F:617 | BSA  (within 1 week） | N/A | Hypertension  D:369 N:424  Atrial Fibrillation  D:141 N:97  Diabetes  D:86 N:108  Glasgow Coma Score <11 D:245 N:16  Black ethnicity  D:73 N:119 | Dysarthia  D:166 N:80 |
| Avinash  2019[39] | *Switzerland*  Europe | 2015.1  2015.12  (12 M) | 75(21-96) | acute ischemic stroke  Anterior circulation  D:63 N:170  Posterior circulation  D:10 N:51  Simultaneous anterior and posterior,D:4 N:9  （Acute） | NIHSS score  0-4.D:10 N:113  5-15 D:39 N:95  ＞15 D:29 N:43 | 81  M:40  F:41 | 340  M:183  F:157 | The Burke dysphagia screening test (within 24h admission) | N/A | Hypertension,  D:65 N:192  Diabetes,D:16 N:52  Hyperlipidemia  D:64 N:202  Current smoking  D:19 N:47  Atrial ﬁbrillation  D:41 N:74  Dental prosthesis  D:13 N:10  Previous clinical stroke (or TIA),  D:14 N:58 | In hospital mortality  D: 7 N:22  Death at 12 month,  D:26 N:49 |
| Polo  2009[40] | *Italy*  Europe | 2005.1-2008.12 | 79.4±6.2  D: 80.7±5.4  N: 78.6±6.6 | Ischemic, D:47 N:65  Hemorrhagic, D:15 N:24  Cortical stroke dominant side  TACI or PACI, D:15 N:18  Cortical stroke nondominant side TACI orPACI, D:20N:15  Subcortical stroke dominant side striatocapsular PACI or LACI, D:12 N:12  Subcortical stroke nondominant side  striatocapsular PACI or LACI, D:4 N:26  Brainstem stroke POCI,  D:8 N:8  Cerebellar stroke POCI,  D:3 N;8  Mixed or multifocal  Stroke, D:0 N:2 | functional independence measurement (FIM) score,  D:35.6±15.6  N:53.1±2.1  and level of cognitive functioning (LCF) score,  6.3±1.3  N:5.7±1.4  N:6.7±1 | 62  M:31  F:31 | 151  M:77  F:74 | BSA  (within 24h from admission) | N/A | Previous Stroke  D:15 N:9 | FIM（discharge）：  D: 46.8 ±24.4  N:68.5±25  Length of stay, days  N:26.6±12.3  D:35±16.7  Lower respiratory tract infection, D:8 N:1  Malnutrition  D:59 N:73  Aphasia, D:32 N:16  Dysarthria,D:55 N:28 |
| Rofes  2018[41] | *Spain*  Europe | 2010.5-  2014.9 | 73.2 ± 13.13 | Ischemic stroke D:161 N:205  Intraparenchymal hemorrhage,D:15 N:10   Subarachnoid hemorrhage  D:1 N:1   Cerebral venous thrombosis  D:1 N:1  Stroke lesion volume (cc)  D:46.8 ± 112.6 N:8.8 ± 21.1 | NIHSS Score  3(1-6) | 178  M:85  F:93 | 395  M:211  F:184 | V-VST  ( between 24 and 48 hours after admis-  sion) | N/A | Hypertension  D:149 N:162   Diabetes mellitus  D:71 N:79   Dyslipidemia  D:105 N:128   Heart disease  D:61 N:49  Major cardioembolic  D:42 N:29  Previous Stroke  D:41 N:32  mRS (score >1)  D:64 N:33 | Dischange: Neurological complications  D: 25 N:10   Respiratory  Infections  D: 21 N:3   Urinary infections  D:12 N:2   Length of stay  (days)  D: 8.2±5 N:6.1±2.9  Mortality D: 20 N:1  3 /12months follow­up:  Readmissions  D:34/75 N:57/117   Respiratory infections  D: 12/45 N:7/28   Mortality  D:41/55 N:3/7 |
| Mohamed2016[42] | *German*  Europe | 2002.10-  2011.3 | 73±13  D:76±12  N:72±13 | acute ischemic stroke  12276  （＜72h） | NIHSS Score  D:13 (7–18)  N: 3 (2–5) | 3083  M:1336  F:1747 | 12276  M:6317  F:5959 | systematic dysphagia  screening clinically  (<3h, N:3503 D:1331  3h to <24h,N:2343 D:1089  24h to ≤72h,N:230 D:165  >72h,N:75 D:55) | N/A | Hypertension  D:2615 N:7552  Diabetes mellitus  D:846 N:2223  Hypercholesterolemia  D:1455 N:5022  Previous stroke  D:1050 N:2405  Atrial fibrillation  D:1404 N:2340 | Aphasia  D:1803 N:2145  Dysarthria  D:2660 N:3066  Unilateral weakness  D;2824 N:5865  Death  D:457 N:106  Disability at discharge (mRS ≥2)  D:2291 N:4628  Length of hospitalization  D:10.5±7 N:9.2±5  3个月随访无数据 |
| Carlo  2019[43] | *Italy*  Europe | 2013-2016 | 72.6±15.5  ≤74 D:41 N:84  ＞74 D:53 N:71 | ischemic stroke  D:72 N:132  Hemorrhagic  D:22 N:23  Hemispheric  D:88 N:143  Brainstem + cerebellar  D:6 N:12  (＜48h) | N/A | 94  M:45  F:49  (7d) | 249  M:126  F:123 | 3-oz water swallow  Test  (7d,30d) | N/A | Renal failure, D:9 N:5  Heart failure,  D:18N:23  Liver failure  D:2 N:2  Antidiabetic therapy  D:28 N:56  Previous stroke  D:13 N:24  mRS, modified Rankin Scale  (0-2)D:16/12 N:74/64  (3-5)D:78/63 N:81/78 | 7days/1 momth(217)  Death  D:3/23 N:10/2  Parica  D:34/22 N:27/16  1 month:  Dysphagia still had  D:75 N:142 |
| Sachiyo  2021[44] | *America*  North America | 2017.12-2018.11  (6 months follow-up） | 71.7±13.4  D:74.5±12.9  N:68.83±13.1 | Ischemic,104  D:70 N:34  Hemorrhagic,323  D:163 N:160  (3.1±4d) | NIHSS score  D:13.3±9.7  N:2.8±2.6  2 (GCS)  D: 13.1±2.7  N:14.9±0.5 | 233  M:141  F:92 | 427  M:266  F:161 | RSST& MWST&  TOR-BSST  （3.1±4d） | Dysphagia Severity Scale, (DSS）  D:mean :4 | Atrial ﬁbrillation  D: 46 N:26  Diabetes mellitus  D;65 N:58  Hypertension  D:189 N:143  Smoking ever  D:66 N:71  Hyperlipidemia  D:81 N:82  Hypercholesterolemia  D:12 N:7  Previous stroke  D:67 N:34 | Discharge：  Dysphagia still had:158  6 months follow-up:  Death:  D:26(233) |
| Håkan  1998[45] | *Sweden*  Europe | N/A | D: 76 ± 6  N: 73 ± 11 | Ischemic,  Hemorrhagic, | N/A | 14  M:7  F:7 | 72  M:24  F:48 | repetitive oral  suction swallow test ( ROSS test)  Within 24 h | N/A | Previous stroke  D:4 N:12  Hypertension  D;7 N:22  Atrial fibrillation  D:3 N:11  Other cardiac disorder  D:3 N:18  Diabetes mellitus  D:3 N:10  Current smoker  D:1 N:7  Facial paresis  D:11 N:22 | Barthel Index day 1  D: 8.8 ± 8.0  N: 9.9 ± 8.4  Barthel Index day 7  D: 10.8 ± 7.8  N: 10.2 ± 7.8  Pneumonia D:2 N:0  Discharge:  Returned home  D:10 N:46  Death D:0 N:1  Days in hospital  D:14(8-47) N10(6-22)  6 month Follow-Up  Dysphagia still had:2 |
| Anna  2012[46] | South  America | 2005.5 – 2006.7 | Mean D:62N:65  ＜60 D:40 N:32  ≥60 D:94 N:46 | Ischemic,177  D:108 N:69  Hemorrhagic,35  D:26 N:9  Undetermined:D:12 N:17 | Barthel index:  Independent:mildly/moderately/severely/totallydepend:  D:6/14/14/15/85  N:21/24/16/6/11  mRS:1/2/3/4/5  D:4/4/10/23/93  N:19/7/13/28/11 | 134  M:79  F:55 | 212  M:125  F:87 | BSA  ＜5days:172 11– 20:14，  21-60:26  （未提及具体） | Mild, 26  Moderate,51  Severe,57 | mRS:1/2/3/4/5  D:4/4/10/23/93  N:19/7/13/28/11  Previous stroke  D:50 N:16 | 3 months follow-up:  Death:D:35 N:4 |
| Aline  2016[47] | South America | N/A | Mean:62.6  D:63.1±16.7  N:62±12.9  （＜48h） | Ischemic,78  Hemorrhagic,22  lacunar (LAC), D:7 N:11  total anterior circulation (TAC), D:23 N:5  partial anterior circulation (PAC), D:7 N:12  posterior circulation (POC)  D:4 N:9 | N/A | 50  M:27  F:23 | 100  D:46  N:54 | Gugging Swallowing Screen (GUSS)  （48h） | Mild, 11  Moderate,11  Severe,28 | Previous stroke  D:14 N:6  Diabetes mellitus  D:11 N:13  HypertensionD:39 N:42  Heart diseaseD:18 N:12  Alzheimer D:1 N:0  Smoking D:12 N:15  Use of alcohol D:12 N:7 | Aphasic D:23 N:4  Dysarthric D:11 N:5 |
| Felix  2021  [48] | *Germany*  Europe | 2015.12-  2018.11 | D:73.1±14.1  N:70.5±14.5 | intracerebral hemorrhages  (Onset to hospital admission <72 hours)  RHD: D:36 N:23  Deep brain ICH, D:37 N:10  Basal ganglia, D:25 N:6  Thalamus, D:26 N:6  Infratentorial, D:10 N:6  Frontal, D:31 N:7  Parietal, D:21 N:18  Temporal, D:10 N:9  Occipital, D:3 N:10 | NIHSS score  D:12(8.3-15）  N:4(1-7)  Volume  D:27.96±27.8  N:12.68±22.3  mRS  D:4(4-5)  N:2(1-4) | 84  M:48  F:36 | 132  M:78  F:54 | speech-language pathologist clinical assessment  (within 48h from admission) | N/A | Previous stroke,  D:14 N:9  Diabetes,  D:14 N:14  Facial palsy,  D:42 N:11  Buccofacial apraxia  D:9 N:1 | Pneumonia,  D:48 N:11  Aphasia  D:19 N:7 |

PSD: Post-stroke dysphagia; M:male; F: female; N: no dysphagia; D:dysphagia; N/A: Not mentioned in article; CNS: Canadian Neurological Scale ; MMSE: Mini-Mental State Examination ; BSA: bedside swallowing assessment ;RSST: repetitive saliva swallowing test; MWST: the modified water swallowing test; WST: water swallowing test ;MASA: Mann Assessment of Swallowing Ability; RHD: Right hemisphere damage; LHD: Left hemisphere damage; NIHSS:National Institutes of Health Stroke Scale.; V-VST:Volume-Viscosity Swallow Test; DOSS: Dysphagia outcome severity scale; VFSS: Videofluoroscopy Assessment；ASHA-NOMS:American Speech-Language-Hearing Association National Outcomes Measurement System swallowing scale level；NDPCS: Northwestern Dysphagia Patient Check Sheet ; GUSS: Gugging Swallowing Screen; FEES: fiberoptic endoscopic evaluation of swallowing ; FEDSS: fiberoptic endoscopic dysphagia severity score ; 3-SSS: 3-step swallowing screen; FOIS: Functional Oral Intake Scale ; PIODA: the Protocol for the Investigation of Oropharyngeal Dysphagia in Adults; ; LACI, lacunar infarction; PACI, partial anterior circulation infarction; POCI, posterior circulation infarction; TACI, total anterior circulation infarction.

MRC: Medical Research Council

**Appendix 2.** **Details of the evaluation scale(NOS & AHQR)**

***1.Newcastle–Ottawa scale (NOS)***

**Case-control studies**

***SELECTION***

1. **Is the Case Definition Adequate?**
2. Requires some independent validation
3. Record linkage
4. No description
5. **Representativeness of the Cases**
6. All eligible cases with outcome of interest over a defined period of time, all cases in a defined catchment area, all cases in a defined hospital or clinic, group of hospitals, health maintenance organisation, or an appropriate sample of those cases
7. Not satisfying requirements in part (a), or not stated.
8. **Selection of Controls**

This item assesses whether the control series used in the study is derived from the same population as the cases and essentially would have been cases had the outcome been present.

1. Community controls
2. Hospital controls, within same community as cases but derived from a hospitalised population
3. No description
4. **Definition of Controls**
5. If cases are first occurrence of outcome, then it must explicitly state that controls have no history of this outcome. If cases have new (not necessarily first) occurrence of outcome, then controls with previous occurrences of outcome of interest should not be excluded.
6. No mention of history of outcome

***COMPARABILITY***

1. **Comparability of Cases and Controls on the Basis of the Design or Analysis**
2. ***EXPOSURE***
3. **Ascertainment of Exposure**
4. **Non-Response Rate**

**Cohort studis**

***SELECTION***

1. **Representativeness of the Exposed Cohort**

Item is assessing the representativeness of exposed individuals in the community, not the representativeness of the sample of women from some general population.

1. **Selection of the Non-Exposed Cohort**
2. **Ascertainment of Exposure**
3. **Demonstration That Outcome of Interest Was Not Present at Start of Study**

***COMPARABILITY***

1. **Comparability of Cohorts on the Basis of the Design or Analysis**

***OUTCOME***

1. **Assessment of Outcome**
2. Independent or blind assessment stated in the paper, or confirmation of the outcome by reference to secure records (x-rays, medical records, etc.)
3. Record linkage
4. Self-report
5. No description.
6. **Was Follow-Up Long Enough for Outcomes to Occur**
7. **Adequacy of Follow Up of Cohorts**

***2.Agency for Healthcare Research and Quality (AHRQ)***

(1) The source of information (survey and record review) was defined. (2) Inclusion and exclusion criteria for exposed and unexposed subjects (cases and controls) were listed or referred to in previous publications. (3) The time period used to identify patients was indicated. (4) An indication of whether subjects were consecutive, if not population-based, was provided. (5) An indication of whether evaluators of subjective components of the study were masked to other aspects of the status of the participants was provided. (6) Assessments undertaken for quality assurance purposes (e.g., test/retest of primary out-of-come measurements) were described. (7) Any patient exclusion from the analysis was explained.(8) Assessment and/or control of confounding factors was described. (9) If applicable, the handling of missing data was described.

(10) Patient response rates and completeness of data collection were summarized. (11) Clarification of what follow-up, if any, was expected and the percentage of patients for whom incomplete data or follow-up was obtained was provided.

A total of 11 points: 0-3, low quality; 4-7, medium quality; and 8-11, high quality.

eTable 4. quality assessment of case control study and cohort studies

| STUDY | SELECTION | COMPAIRABILITY | OUTCOME | TOTAL |
| --- | --- | --- | --- | --- |
| Marlis 2008 | 4 | 2 | 0 | 6 |
| Antía 2019 | 2 | 0 | 2 | 4 |
| Hamidon 2006 | 2 | 1 | 3 | 6 |
| Michael 2012 | 2 | 2 | 2 | 6 |
| Giselle 2000 | 2 | 1 | 3 | 6 |
| Maurizio 2004 | 4 | 2 | 3 | 9 |
| Anna 201717 | 2 | 1 | 1 | 4 |
| Heather 2017 | 3 | 1 | 1 | 5 |
| Sani 2017 | 2 | 1 | 2 | 5 |
| Bendix 2018 | 2 | 1 | 1 | 4 |
| Zeki 2010 | 4 | 1 | 3 | 8 |
| Kaila 2019 | 3 | 0 | 1 | 4 |
| Shiva 2019 | 3 | 1 | 1 | 5 |
| Elien 2020 | 2 | 1 | 1 | 4 |
| Nayeon 2021 | 2 | 1 | 1 | 4 |
| Juli 2019 | 2 | 1 | 3 | 6 |
| Healther 2013 | 2 | 1 | 1 | 4 |
| Smtthards 2007 | 2 | 1 | 3 | 6 |
| Avinash 2019 | 2 | 1 | 2 | 5 |
| Polo 2009 | 2 | 0 | 1 | 3 |
| Rofes 2018 | 2 | 1 | 3 | 6 |
| Mohamed 2016 | 2 | 1 | 3 | 6 |
| Carlo 2019 | 2 | 1 | 3 | 6 |
| Sachiyo 2021 | 3 | 1 | 3 | 7 |
| Håkan 1998 | 3 | 1 | 2 | 6 |
| Anna 2012 | 4 | 1 | 3 | 8 |
| Felix 2021 | 2 | 1 | 1 | 4 |

eTable 5. quality assessment of Cross-section studies

| AUTHOR& YEAR | Item1 | Item2 | Item3 | Item4 | Item5 | Item6 | Item7 | Item8 | Item9 | Item10 | Item11 | Scores |
| --- | --- | --- | --- | --- | --- | --- | --- | --- | --- | --- | --- | --- |
| Zahra 2021 | 1 | 1 | 1 | 0 | 1 | 1 | 0 | 0 | unclear | 0 | unclear | 5 |
| Eman 2021 | 1 | 1 | 1 | 0 | 1 | 1 | 1 | 0 | 0 | 1 | unclear | 7 |
| Mahsa 2021 | 1 | 1 | 1 | 0 | 1 | 1 | 0 | 0 | 0 | 1 | unclear | 6 |
| SK 2017 | 1 | 1 | 1 | 1 | 1 | 1 | 0 | 0 | 0 | 1 | unclear | 7 |
| Danielle 2016 | 1 | 1 | 1 | 1 | 1 | 1 | 1 | 0 | unclear | 1 | unclear | 7 |
| Shiva 2016 | 1 | 1 | 1 | 1 | 1 | 1 | 1 | 0 | 0 | 1 | unclear | 8 |
| Alien 2016 | 1 | 1 | 1 | 1 | 1 | 1 | 0 | unclear | unclear | 1 | unclear | 7 |

eFigure 1. Overall prevalence of psd

eFigure 2. Subgroup analysis of prevalence of psd（by type of stroke）

eFigure 3. Subgroup analysis of prevalence of psd（by sex）

eFigure 4. Subgroup analysis of prevalence of psd（by stroke history）

eFigure 5. Subgroup analysis of prevalence of psd（by diagnose time）

eFigure 6. Subgroup analysis of prevalence of psd（by stroke lesion）

eFigure 7. Subgroup analysis of prevalence of psd（by diagnose method）

eFigure 8. Subgroup analysis of prevalence of psd（by continents）

DH: PSD with hypertension ; DnH: PSD without hypertension; NH: Non-psd with hypertension; NnH: Non-psd without hypertension.

eFigure 9. Risk factor analysis (hypertension)

DD: PSD with diabetes ; DnD: PSD without diabetes; ND: Non-psd with diabetes; NnD: Non-psd

without diabetes.

eFigure 10. Risk factor analysis (Diabetes)

DS: PSD with smoking ; DnS: PSD without smoking; NS: Non-psd with smoking; NnS: Non-psd without smoking.

eFigure 11. Risk factor analysis (Smoking)

DP: PSD with previous stroke ; DnP: PSD without previous stroke; NP: Non-psd with previous stroke; NnP: Non-psd without previous stroke.

eFigure 12. Risk factor analysis (Previous stroke)

DTIA: PSD with previous TIA ; DnTIA: PSD without previous TIA; NTIA: Non-psd with previous TIA; NnTIA: Non-psd without previous TIA.

eFigure 13. Risk factor analysis (Previous TIA)

DAF: PSD with atrial fibrillation ; DnAF: PSD without atrial fibrillation; NAF: Non-psd with atrial fibrillation; NnAF: Non-psd without atrial fibrillation.

eFigure 14. Risk factor analysis (Atrial fibrillation)

DHD: PSD with heart disease ; DnHD: PSD without heart disease; NHD: Non-psd with heart disease; NnHD: Non-psd without heart disease.

eFigure 15. Risk factor analysis (Heart disease)

DDy: PSD with dyslipidemia ; DnDy: PSD withoutdyslipidemia; NDy: Non-psd with dyslipidemia; NnDy: Non-psd without dyslipidemia.

eFigure 16. Risk factor analysis (Dyslipidemia)


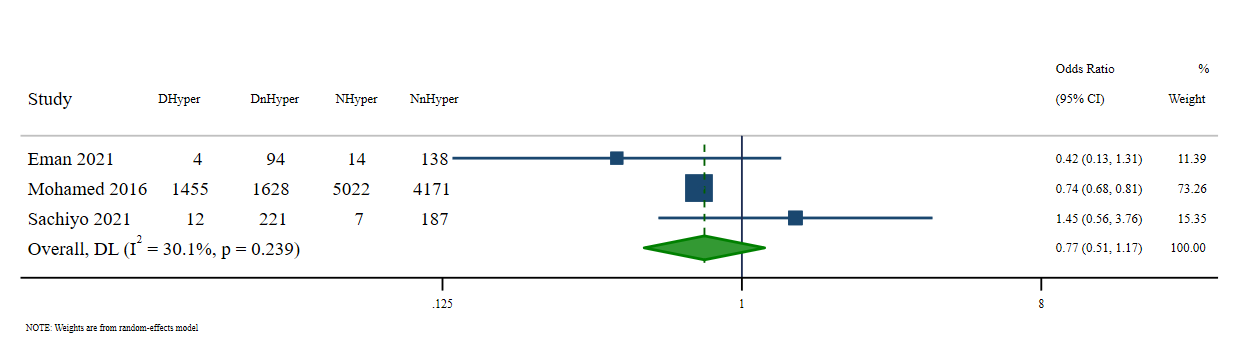


DHyper: PSD with hypercholesterolemia ; DnHyper: PSD without hypercholesterolemia; NHyper: Non-psd with hypercholesterolemia; NnHyper: Non-psd without hypercholesterolemia.

eFigure 17. Risk factor analysis (hypercholesterolemia)

DHyper: PSD with obesity ; DnHyper: PSD without obesity; NHyper: Non-psd with obesity; NnHyper: Non-psd without pbesity.

eFigure 18. Risk factor analysis (Obesity)

N:non-psd

eFigure 19. Outcome analysis (Prevalence of aphasia in psd and non-psd)

N:non-psd

eFigure 20. Outcome analysis (Prevalence of dysarthria in psd and non-psd)

N:non-psd

eFigure 21. Outcome analysis (Prevalence of respiratory tract infection in psd and non-psd)

N:non-psd

eFigure 22. Outcome analysis (Prevalence of pneumonitis in psd and non-psd)

N:non-psd

eFigure 23. Outcome analysis (Prevalence of dysphagia persists in psd )

N:non-psd

eFigure 24. Outcome analysis (Mortality rate in psd )

**Appendix 3. Publication Bias**

Funnel chart

Publication bias of prevalence studies of dysphagia after stroke based on Regression-based Egger test

----------------------------------------------------------------------------------------------------------------------

_meta_es | Coef. Std. Err. z P>|z| [95% Conf. Interval]

----------------------------------------------------------------------------------------------------------------------

_meta_se | .9243697 1.728693 0.53 0.593 -2.463807 4.312546

_cons | .4316892 .0709767 6.08 0.000 .2925775 .5708009

Table 6.Meta-regression analysis of heterogeneity introduced by study characteristics

| Variable | Coefficient | SE | t | p> \|t\| | 95% CI |
| --- | --- | --- | --- | --- | --- |
| Public year | 0.003 | 0.006 | 0.50 | 0.620 | (-0.011 to 0.017) |
| Continents | 0.339 | 0.019 | 1.82 | 0.078 | (-0.004 to 0.072) |
| Study design | -0.881 | 0.062 | -1.43 | 0.164 | (-0.214 to 0.039) |
| Diagnose method | 0.265 | 0.064 | 4.12 | ＜0.001 | (0.134 to 0.396） |


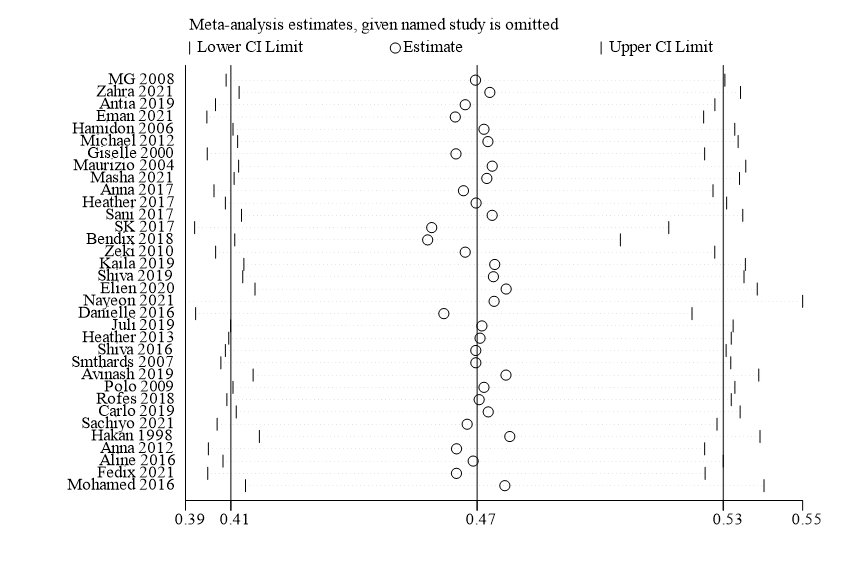


eFigure 25. Sensitivity analysis
